# Modeling gene × environment interactions in PTSD using human neurons reveals diagnosis-specific glucocorticoid-induced gene expression

**DOI:** 10.1038/s41593-022-01161-y

**Published:** 2022-10-20

**Authors:** Carina Seah, Michael S. Breen, Tom Rusielewicz, Heather N. Bader, Changxin Xu, Christopher J. Hunter, Barry McCarthy, P. J. Michael Deans, Mitali Chattopadhyay, Jordan Goldberg, Saunil Dobariya, Frank Desarnaud, Iouri Makotkine, Janine D. Flory, Linda M. Bierer, Migle Staniskyte, NYSCF Global Stem Cell Array® Team, Lauren Bauer, Katie Brenner, Geoff Buckley-Herd, Sean DesMarteau, Patrick Fenton, Peter Ferrarotto, Jenna Hall, Selwyn Jacob, Travis Kroeker, Gregory Lallos, Hector Martinez, Paul McCoy, Frederick J. Monsma, Dorota Moroziewicz, Reid Otto, Kathryn Reggio, Bruce Sun, Rebecca Tibbets, Dong Woo Shin, Hongyan Zhou, Matthew Zimmer, Scott A. Noggle, Laura M. Huckins, Daniel Paull, Kristen J. Brennand, Rachel Yehuda

**Affiliations:** 1 Pamela Sklar Division of Psychiatric Genomics, Department of Psychiatry or Department of Genetics and Genomic Sciences, Icahn School of Medicine at Mount Sinai, New York, NY USA; 2 Nash Family Department of Neuroscience or Friedman Brain Institute, Icahn School of Medicine at Mount Sinai, New York, NY USA; 3 Departments of Psychiatry and Genetics, Division of Molecular Psychiatry, Yale University School of Medicine, New Haven, CT USA; 4 The New York Stem Cell Foundation Research Institute, New York, NY USA; 5 James J. Peters Veterans Affairs Medical Center, Bronx, NY USA; 6 Center for Psychedelic Psychotherapy and Trauma Research, Icahn School of Medicine at Mount Sinai, New York, NY USA

**Keywords:** Gene expression profiling, Post-traumatic stress disorder

## Abstract

Post-traumatic stress disorder (PTSD) can develop following severe trauma, but the extent to which genetic and environmental risk factors contribute to individual clinical outcomes is unknown. Here, we compared transcriptional responses to hydrocortisone exposure in human induced pluripotent stem cell (hiPSC)-derived glutamatergic neurons and peripheral blood mononuclear cells (PBMCs) from combat veterans with PTSD (*n* = 19 hiPSC and *n* = 20 PBMC donors) and controls (*n* = 20 hiPSC and *n* = 20 PBMC donors). In neurons only, we observed diagnosis-specific glucocorticoid-induced changes in gene expression corresponding with PTSD-specific transcriptomic patterns found in human postmortem brains. We observed glucocorticoid hypersensitivity in PTSD neurons, and identified genes that contribute to this PTSD-dependent glucocorticoid response. We find evidence of a coregulated network of transcription factors that mediates glucocorticoid hyper-responsivity in PTSD. These findings suggest that induced neurons represent a platform for examining the molecular mechanisms underlying PTSD, identifying biomarkers of stress response, and conducting drug screening to identify new therapeutics.

## Abstract (web summary)

The authors generated stem cell-derived neurons from combat veterans with and without PTSD and found PTSD-dependent gene expression changes in response to glucocorticoids. This highlights how stress response may be altered in individuals with PTSD.

## Main

Although unequivocally precipitated by environmental events, PTSD develops in only a minority of trauma survivors^1^, prompting a search for risk factors that increase the probability of developing this condition following trauma exposure. Convergent lines of evidence are consistent with a heritable component to PTSD risk. There is a concordance between PTSD diagnosis in monozygotic and dizygotic twins^2^. Genome-wide association studies (GWAS) estimate single nucleotide polymorphism (SNP)-based heritability from 5% to 30%^3–5^ and identify loci significantly associated with PTSD^3,5^, although loci vary with ancestry, sex and type of trauma. This highlights the necessity of developing paradigms that examine the impact of PTSD genetic risk factors in the context of exposure to an environmental stressor.

Insights into the gene by environment (G×E) interactions underlying the psychiatric symptoms of PTSD remain poorly resolved, reflecting the lack of a cohesive neurobiological framework to investigate these mechanisms. To date, most studies of PTSD pathophysiology have focused on PBMCs, with PTSD patients showing lower ambient cortisol and heightened glucocorticoid sensitivity relative to healthy controls^6,7^ coupled with increased expression of innate immune genes^8,9^. Toward resolving the relative contributions of genetic risk and environmental stress to PTSD pathophysiology, it is critical to deconvolve the impact of stress in a cell-specific manner, including determinants of stress responses across brain and blood cells. Glucocorticoid receptor signaling is the most overlapping pathway between murine blood and brain and is highly associated with individual differences in response to trauma exposure^10^. The degree to which PTSD symptoms arise as a result of elevated/sustained peripheral response to stress^6,10^ and/or abnormal cellular or circuit response to peripheral stress cues^11–13^ remains unresolved. In either case, preclinical data suggests that trauma-induced perturbations in glucocorticoid signaling result in glutamate-induced excitotoxicity, leading to decreased glutamatergic neural activity, dendritic retraction and reduced synaptic density^14^. Drugs that modulate the glucocorticoid^15,16^ and glutamatergic^17^ systems represent potential avenues for pharmacologic intervention.

An improved understanding of the pathophysiology of PTSD requires the development of appropriate human-specific models. Understanding the extent to which the dysregulated stress response reflects cell-type-specific preexisting genetic vulnerabilities will improve genetic-based diagnosis and potentially identify new therapeutic targets to prevent or reverse PTSD. hiPSC-based models have the potential to address some of the main limitations of clinical and animal studies, evaluating human glutamatergic neurons that are genetically identical to those of their donors (reviewed in Fernando et al.^18^). Although there have been a handful of studies of glucocorticoid response in live human neurons^19,20^, none have contrasted neurons derived from trauma-exposed controls and PTSD cases.

Here, we describe a hiPSC-based study of PTSD, comparing glutamatergic neurons from combat veterans with (*n* = 19 donors) and without (*n* = 20 donors) PTSD, to test the hypothesis that PTSD risk variants and glucocorticoid response (for example, to dexamethasone (DEX) and hydrocortisone (HCort)^21,22^) interact in a cell-type-specific manner. Transcriptional profiles of hiPSC neurons were compared with a well-matched and largely overlapping PBMC study of combat veterans with (*n* = 20 donors) and without (*n* = 20 donors) PTSD. First, glucocorticoid-induced blood and neuronal responses were significantly enriched for immune response, brain development and neurodevelopmental disorder genes, with specific upregulation of disorder genes in neurons only. Second, a baseline PTSD diagnosis-specific signature was undetectable in either human neurons or PBMCs. Third, glucocorticoid hypersensitivity occurred in samples from PTSD cases, with diagnosis-specific effects greatest at low doses, and significantly more robust in neurons than PBMCs. This PTSD-specific neuronal glucocorticoid-response signature was enriched for transcriptomic patterns observed in postmortem (PM) brain tissue from PTSD cases. Critical aspects of glucocorticoid response are encoded in patient genetics, consistent with a clear genetic predisposition to PTSD. These findings suggest possible therapeutic strategies to minimize the likelihood of PTSD following trauma exposure, a particularly valuable outcome for military and first-responder personnel.

## Results

To study how glucocorticoids influence gene expression in donor-specific brain and blood cells, skin and blood samples were collected from well-matched and largely overlapping (30 shared individuals) hiPSC-derived glutamatergic neuron and PBMC cohorts respectively, comprised of combat veterans with (*n* = 19 hiPSC donors and *n* = 20 PBMC donors) and without (*n* = 20 hiPSC donors and *n* = 20 PBMC donors)) PTSD (Supplementary Table 1). For technical reasons, glucocorticoid treatment of *NGN2*-neurons (batch 1 *n* = 15 versus 15, batch 2 *n* = 4 versus 5) and PBMCs (batch A *n* = 10 versus 10, batch B *n* = 10 versus 10 (ref. ^23^)) were completed independently and then meta-analyzed together to adjust for batch effects (Supplementary Table 2).

### Dexamethasone-stimulated transcriptional responses in PBMCs

**Fig. 1:**
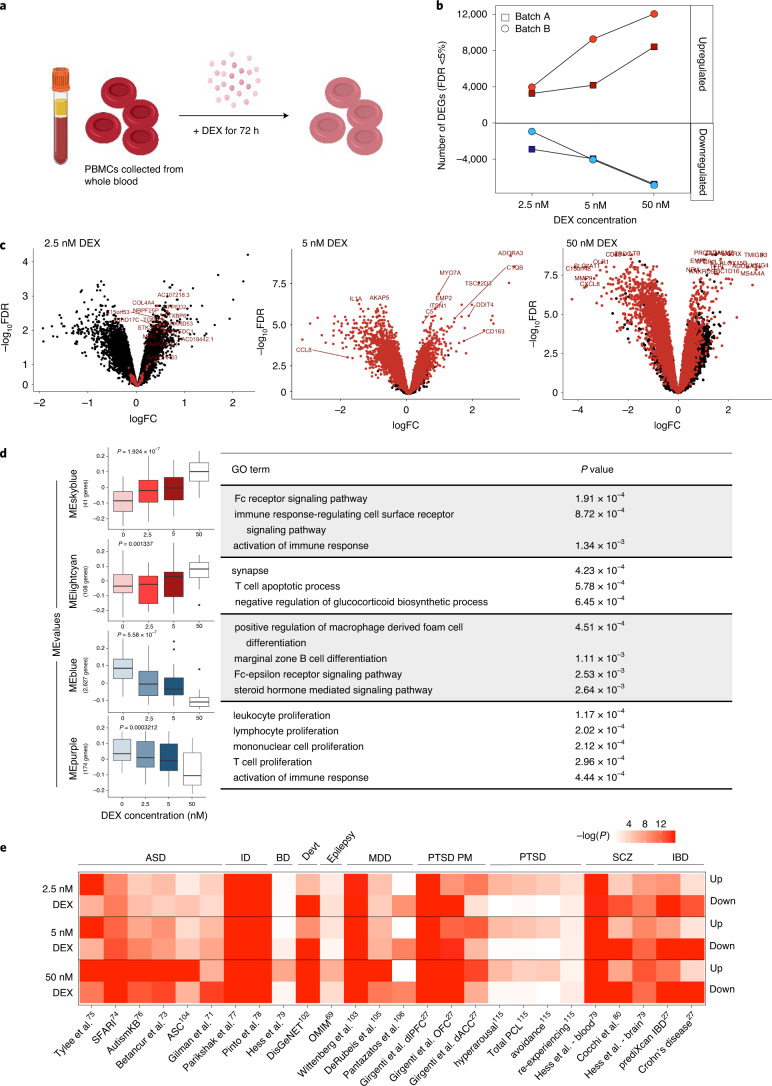
Transcriptional response to DEX in PBMCs. **a**, PBMCs from 20 PTSD cases and 20 combat-exposed controls were treated with DEX for 72 h and RNA-seq was performed. **b**, The number of DEGs observed in batch A (*n* = 10 versus 10) (squares) and batch B (*n* = 10 versus 10) (circles) are upregulated and downregulated (*y* axis) across three different concentrations of DEX conditioning (*x* axis) at a Bonferroni-corrected *P* value threshold. **c**, Meta-analysis of expression logFC (differences observed between vehicle and DEX exposure) was plotted against –log(FDR) for each gene. Red points indicate significantly DEGs in the meta-analysis. Sample-size-based meta-analysis was conducted using METAL with a Cochran’s *Q* test for heterogeneity. Benjamini–Hochberg adjusted *P* values are reported to correct for multiple testing. **d**, ME values from modules identified by WGCNA were correlated with increasing DEX concentrations for all DEX-treated samples (*n* = 40 individuals). Top correlated modules with DEX concentration are shown here (*P* values are labeled above each boxplot). Correlation *P* values were derived from a two-tailed *t* distribution. Data are presented as minimum, first quartile, median, third quartile and maximum. Each module was subjected to GO enrichment analysis and the topmost significant enrichment terms and their associated Benjamini–Hochberg adjusted *P* values are displayed. **e**, Gene set enrichment of DEX-dependent DEGs across psychiatric disorder and neurodevelopmental gene sets^27^. Devt, genesets involved in neurodevelopmental disorders.

Treatment of PBMCs in vitro with the synthetic glucocorticoid DEX is a promising method to explore molecular responses to stress hormones in PTSD^23^. To continue these earlier findings, identical methods were applied to expand this study of combat-exposed veterans to (*n* = 20) and without (*n* = 20) PTSD (Fig. 1a) (Supplementary Tables 1 and 2).

RNA-sequencing (RNA-seq) was generated from cultured PBMCs treated for 72 h with three concentrations of DEX (2.5 nM, 5 nM and 50 nM), and analyzed relative to baseline samples. To identify reliable markers of glucocorticoid activation independent of PTSD, diagnosis as well as other confounds were adjusted for (Methods). Incubation with increasing concentrations of DEX (2.5 nM, 5 nM and 50 nM, respectively) identified 6,153, 8,114 and 15,128 differentially expressed genes (DEGs) in batch A, and 4,880, 13,297 and 18,856 DEGs in batch B, respectively (*Q* value < 0.05) (Fig. 1b and Supplementary Table 4). Overall transcriptome-wide concordance of DEX-induced fold changes (FC) relative to vehicle between the two batches was exceedingly high (average *r* = 0.738) (Extended Data Fig. 5a), and was highly concordant with reported responses to DEX in PBMCs^23^ (*r* = 0.7 in the 50 nM dose) (Extended Data Fig. 5c,d). These findings demonstrate strong conservation of transcriptional changes in PBMCs in response to DEX that are independent of PTSD and other potentially confounding factors.

Gene coexpression modules were calculated to probe functional consequences of DEX exposure in PBMCs. Of 36 significant coexpression modules (M), 24 were significantly dynamically regulated in response to increasing concentrations of DEX (Supplementary Table 5). Gene ontology (GO) analysis found enrichment in neuronal regulation terms such as synapse (module eigengene (ME) lightcyan, *P* = 4.23 × 10^–4^) and immune regulation terms such as activation of immune response (ME skyblue, *P* = 1.34×10^–3^) and lymphocyte proliferation (ME purple, *P* = 2.02 × 10–4), and glucocorticoid signaling terms such as steroid hormone mediated signaling pathway (ME blue, *P* = 2.64 × 10^–3^) and Fc receptor signaling pathway (ME skyblue, *P* = 1.91 × 10^–4^) (Fig. 1d). These enrichments are representative of shared regulation of genes involved in cell migration, cytoskeletal and cell-adhesion properties, which are broadly shared by immune and neural cells, through common processes such as cell migration, degranulation and cellular outgrowth, which may explain the neural term represented in this module. Together, these enrichments are consistent with known glucocorticoid response impacts on cell signaling^24^, neurogenesis^25^ and immunosuppression^26^ (Fig. 1d). Gene set enrichment for psychiatric disorder gene sets^27^ revealed enrichment of DEX-upregulated PBMC DEGs broadly across neuropsychiatric disorder risk genes, but not across PTSD-specific signatures (Fig. 1e). This may indicate that. after adjusting for diagnosis, glucocorticoid treatment of PBMCs alone is insufficient to recapitulate PTSD signatures. Although glucocorticoid treatment of PBMCs (batch A) was highly correlated to the DEX-induced gene responses previously reported (batch B)^23^ (Extended Data Fig. 5a), the larger meta-analysis did not identify PTSD-specific DEX-induced differential response genes (PBMC-DRGs) at a false discovery rate (FDR)-corrected threshold (Extended Data Fig. 9).

### HCort-induced transcriptional responses in neurons

**Fig. 2:**
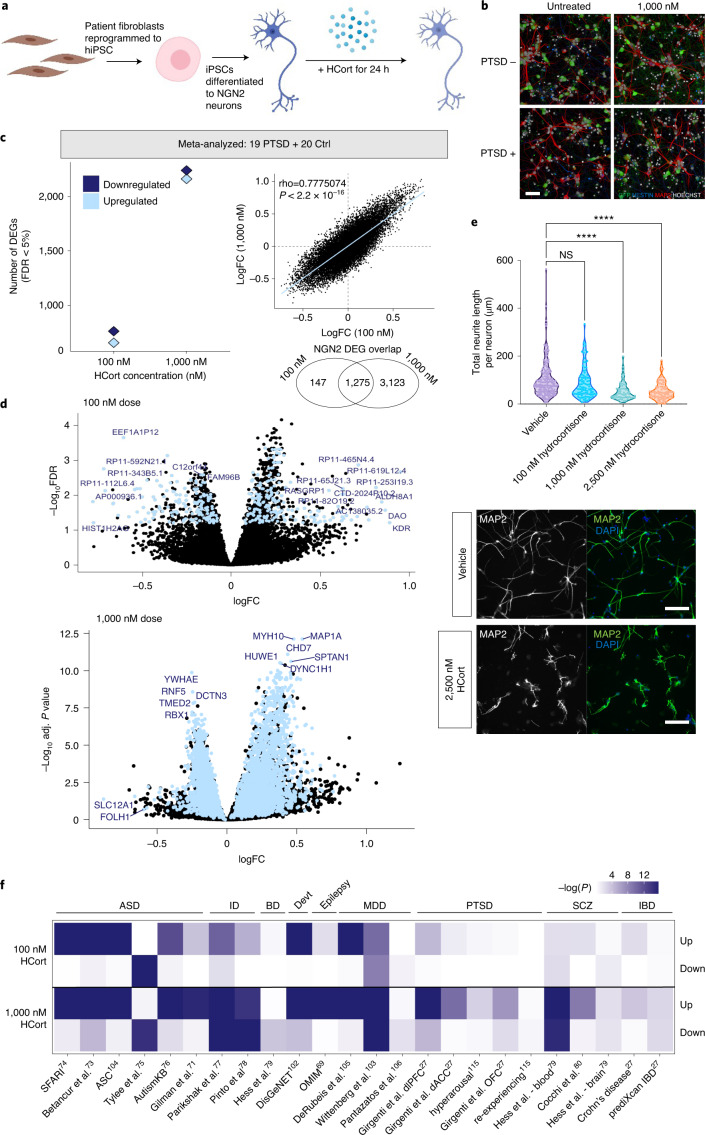
Gene expression changes to HCort in hiPSC-derived neurons. **a**, hiPSC-derived *NGN2*-neurons were treated with HCort for 24 h and RNA-seq performed. **b**, *NGN2*-neurons stained for neuronal markers NESTIN and MAP2, nucleic marker HOECHST and green fluorescent protein to confirm neuronal identity and morphology across all conditions. **c**, Meta-analyzed DEGs in response to increasing concentrations of HCort shows robust changes in *NGN2-*neurons. A comparative analysis of transcriptome-wide log_2_FC in response to different concentrations of HCort in *NGN2-*neurons shows similar responses, indicating a conserved response across all donors to HCort in *NGN2-*neurons. Sample-size-based meta-analysis was conducted using METAL with a Cochran’s *Q* test for heterogeneity. Benjamini–Hochberg adjusted (adj.) *P* values are reported to correct for multiple testing. **d**, Meta-analysis of expression LogFC (differences observed between vehicle and HCort exposure) was plotted against –log(*P* value) for each gene. Blue points indicate significantly DEGs in the meta-analysis. **e**, Morphological analysis of neurite outgrowth in day 7 *NGN2-*neurons showing dose-dependent decrease in neurite outgrowth with HCort exposure. A Kruskal–Wallis test with a post hoc Dunn’s multiple comparisons test was performed on data for neurite length per neuron. **** *P* < <0.0001. NS, nonsignificant. *P* value for the NS comparison was 0.2121. Representative images of neurite morphology to HCort exposure shown below. **f**, Gene set enrichment of HCort-dependent DEGs across psychiatric disorder and neurodevelopmental gene sets^27^.

We^28–31^ and others^32–39^ have demonstrated that *NGN2*-neurons are greater than 95% pure glutamatergic neurons, robustly express glutamatergic genes, release neurotransmitters, produce spontaneous synaptic activity and recapitulate the impact of psychiatric disease-associated genes. Well-matched to the PBMCs described above, 21-day-old *NGN2*-neurons from combat-exposed veterans with (*n* = 19) and without (*n* = 20) PTSD (Supplementary Table 1) were treated with two to three distinct concentrations of HCort (100 nM, 1,000 nM or 2,500 nM) and an untreated baseline condition for 24 h before RNA-seq (Fig. 2a).

**Extended Data Fig. 1:**
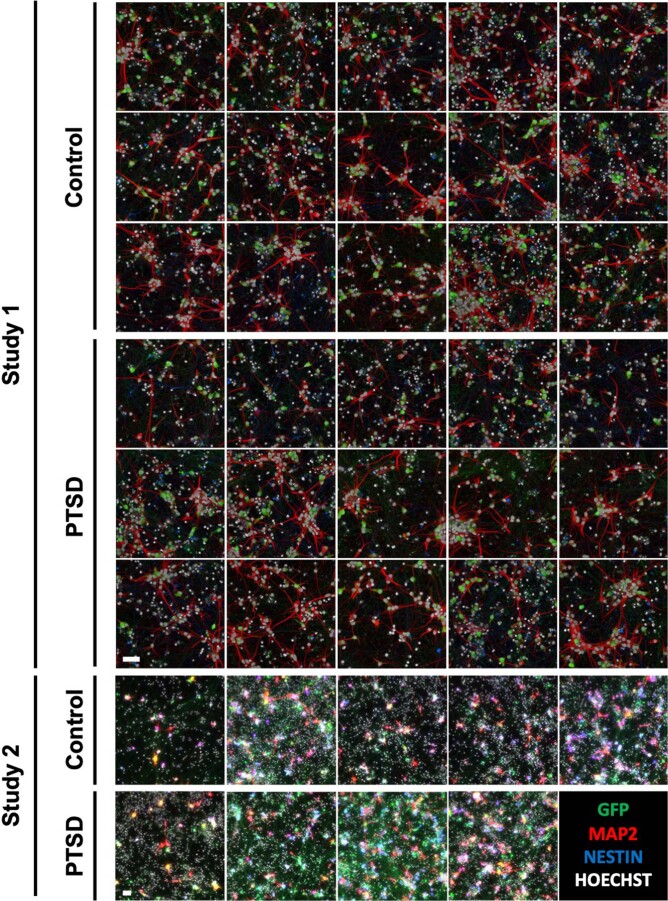
Immunostaining of hiPSC-derived *NGN2-*neurons. Immunostaining of Hoechst (white), GFP (green), MAP2 (red) and NESTIN (blue) across all participants prior to HCort treatment. Imaging was repeated after treatment with the 100 and 1000 HCort dosages. Study 1 and 2: Scale bar = 50 µm.

To confirm the neuronal identity and developmental specificity of these data, a large comparative and integrative analysis was performed across 16 existing transcriptome datasets including 2,716 independent samples from a range of hiPSC-derived neurons and developmentally distinct PM brain tissue^40^. A high degree of transcriptomic convergence was observed for our hiPSC-derived neurons with fetal brain tissue and hiPSC-derived neurons described in previous reports, confirming their early developmental gene expression profiles (Extended Data Fig. 3). Neuronal fate was further confirmed by demonstrated expression of pan-neuronal and synaptic genes^36^ in hiPSC-derived neurons but not in PBMCs (Extended Data Fig. 4a) and VGLUT expression in *NGN2*-neurons (Extended Data Fig. 4b). Glucocorticoid and mineralocorticoid receptor expression was additionally confirmed for each cell type (Extended Data Fig. 4c), with PBMCs demonstrating higher expression of both receptors, consistent with heightened glucocorticoid transcriptional response in PBMCs. Immunostaining of a parallel well demonstrated consistent cell number (4,307 ± 1,313) and neuronal marker expression (78.5 ± 6% microtubule-associated protein 2 (MAP2)-positive and 0.4 ± 0.6% NESTIN-positive) (Fig. 2b and Extended Data Fig. 1) that did not differ significantly by diagnosis or glucocorticoid treatment.

HCort dose-dependent transcriptional responses were resolved relative to those in baseline untreated *NGN2*-neurons. To identify reliable markers of glucocorticoid activation independent of PTSD, diagnosis as well as other confounds were adjusted for. Incubation with increasing HCort concentrations (batch 1: 0 nM, 100 nM and 1,000 nM; batch 2: 0 nM, 100 nM, 1,000 nM and 2,500 nM) resulted in 1,031 and 4,175 DEGs in batch 1, and 165, 3,785 and 4,025 DEGs in batch 2, respectively (*Q* value < 0.05) (Fig. 2c and Extended Data Fig. 6a). Transcriptome-wide concordance was examined using HCort-associated log_2_ FC and HCort responses between doses was highly concordant and preserved across increasing concentrations of HCort in both batches (Extended Data Fig. 6a). Meta-analysis of differential expression summary statistics found 1,837 significant response genes in the 100 nM dose and 5,956 in the 1,000 nM dose (Fig. 2d). To assess whether this large transcriptional response to HCort elicited nonspecific cell toxicity, we measured cell density of untreated and HCort-treated neurons by quantifying the number of Hoescht positive neurons in untreated and treated neurons. We observed no significant differences in cell density between doses (Extended Data Fig. 6b). This suggests that HCort treatment in our neurons did not induce significant cell toxicity. As with PBMCs, gene set enrichment for psychiatric disorder gene sets^27^ revealed enrichment of HCort upregulated *NGN2*-neuron DEGs broadly across neuropsychiatric disorder risk genes, but not across PTSD-specific signatures (Fig. 2f), again suggesting that glucocorticoid treatment of *NGN2*-neurons, without considering PTSD diagnosis, is insufficient to recapitulate PTSD signatures.

**Fig. 3:**
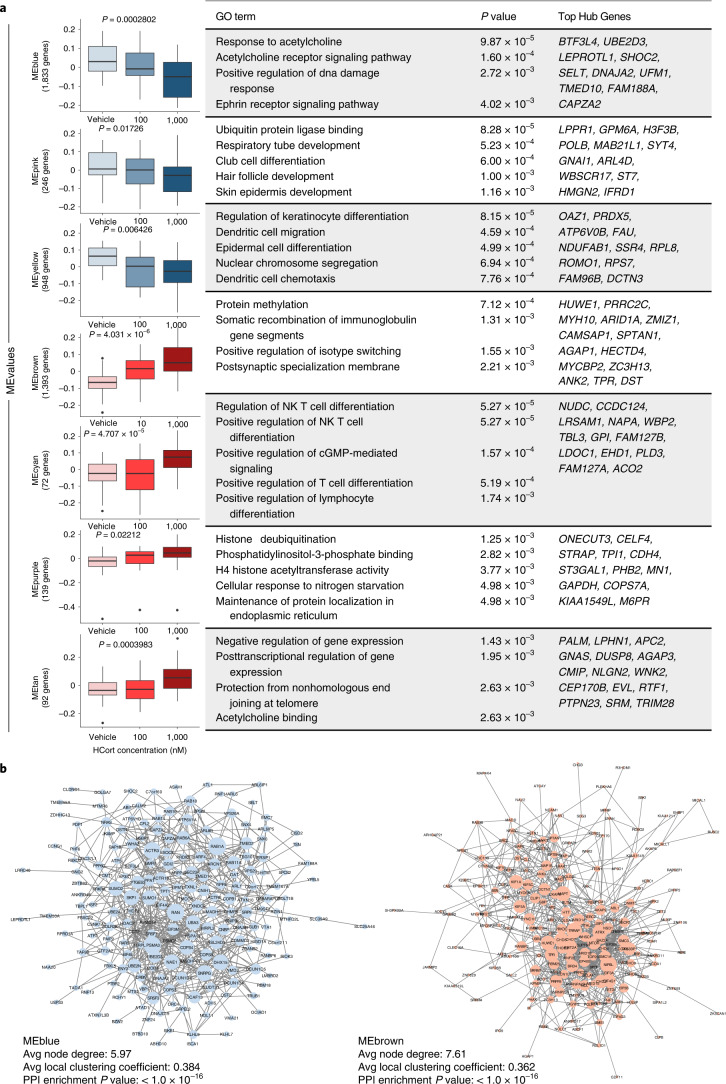
HCort stimulated coexpression modules in *NGN2-*neurons. **a**, WGCNA identified three groups of coregulated gene modules. Pearson correlation was used to assess changes in ME values with increasing concentration of HCort (*P* values are labeled above each boxplot). Correlation *P* values were derived from a two-tailed *t* distribution. Data are presented as minimum, first quartile, median, third quartile and maximum. Each module was subjected to GO enrichment analysis and the topmost significant enrichment terms and their associated Benjamini–Hochberg adjusted *P* values are displayed. Top hub genes (kME > 0.6) within each module are displayed for quick interpretation of GR-stimulated gene coexpression modules and candidate individual genes. **b**, Network visualization of PPIs within modules indicating clusters and network hubs. STRING analysis was used to identify the observed number of edges. Enrichment of observed edges was assessed against expected edges to determine a PPI *P* value of < 1.0 × 10^–16^ for each network. Avg, average.

Weighted gene coexpression modules were calculated from the meta-analyzed studies to better understand the functional aspects of the HCort-induced transcriptional changes in *NGN2*-neurons (Extended Data Fig. 7). Seven significant coexpression modules (M1–7) were identified that were dynamically regulated in response to increasing concentrations of HCort in our signed analysis (Fig. 3a). ME values for M1–3 were downregulated with increasing concentrations of HCort and enriched for biological processes related to acetylcholine signaling, such as acetylcholine receptor signaling pathway (*P* = 1.60 × 10^–4^), protein degradation such as ubiquitin protein ligase binding (*P* = 8.28 × 10^–5^) and skin regulation such as regulation of keratinocyte differentiation (*P* = 8.15 × 10^–5^). These signatures are consistent with known glucocorticoid inhibition of acetylcholine signaling^41^ and role in skin atrophy^42^. Glucocorticoid-acetylcholine signaling interactions occur in pathways affecting memory consolidation^43^, and alter glutamatergic synapses and synaptic stability^44^, suggesting that glucocorticoid exposure alters acetylcholine signaling to impact glutamatergic synaptic biology. Remaining ME values for modules M4–7 significantly increased with HCort treatment. Many terms within these modules enriched for immune processes such as positive regulation of isotype switching (*P* = 1.55 × 10^–3^), regulation of NK T cell differentiation (*P* = 5.27 × 10^–5^), and somatic recombination of immunoglobulin gene segments (*P* = 1.31 × 10^–3^), processes that are well-established markers of glucocorticoid activation^8,9^. Finally, enrichments for hallmark glucocorticoid processes of histone acetylation^45^, such as H4 histone acetyltransferase activity (*P* = 3.77 × 10^–3^) and transcriptional suppression (negative regulation of gene expression (*P* = 1.43 × 10^–3^)), were enriched. Unsigned modules significantly associated with neural projection terms, such as neural crest cell differentiation (*P* = 3.96 × 10^–4^) and regulation of neuron projection development (*P* = 1.04 × 10^–3^), and immune terms, such as regulation of acute inflammatory response (*P* = 1.83 × 10^–3^) (Extended Data Fig. 8). To visualize module connectivity, protein–protein interactions (PPIs) of genes within modules were mapped, finding a significant observed number of edges in all modules (*P* < 1.0 × 10^–16^) (Fig. 3b). Overall, HCort treatment of *NGN2-*neurons, independent of diagnosis, resulted in robust downregulation of acetylcholine signaling and skin development, and upregulation of inflammation-modulating and cell-signaling genes.

As transcriptomic responses to HCort exposure enriched in neuronal projection and signaling terms, we sought to validate these functional measures of HCort exposure in *NGN2*-neurons. To visualize the effects of HCort exposure on neurite outgrowth, we performed neurite tracing analysis on *NGN2-*neurons treated with HCort, finding a dose-dependent decrease in neurite length per neuron after stimulation with 100 nM, 1,000 nM and 2,500 nM of HCort (Fig. 2e). This finding is concordant with previously described dendritic retraction phenotypes in response to trauma-related glucocorticoid signaling^46^.

### PTSD diagnosis-dependent differences in human neurons

**Fig. 4:**
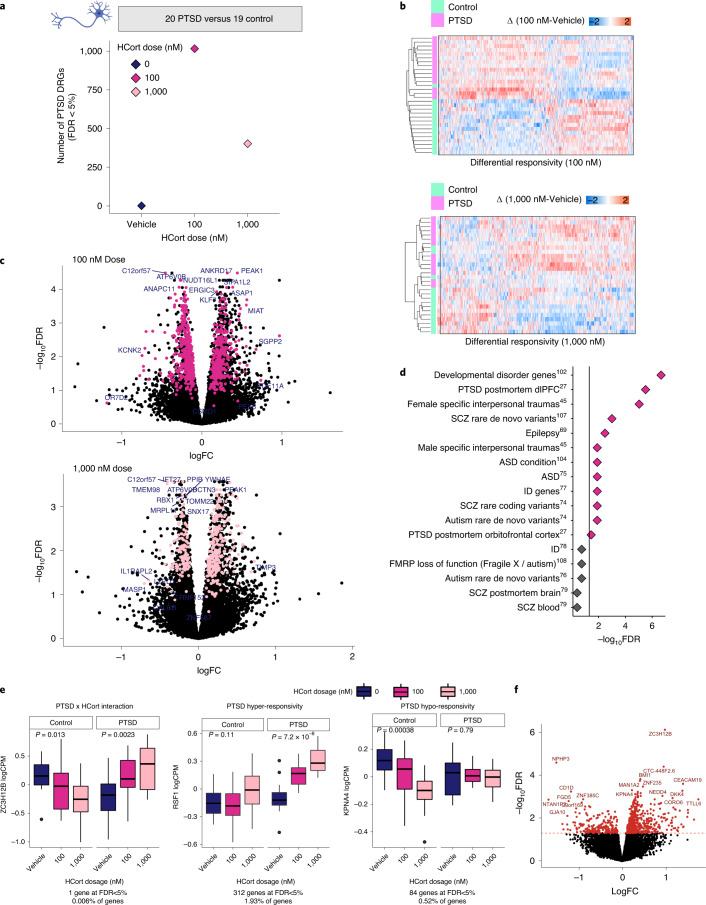
PTSD(+) specific responses to HCort in *NGN2-*neurons. **a**, Genes that differ in their response to HCort in PTSD(+) donors compared with PTSD(–) donors, here termed DRGs, were detected in both the 100 nM and 1,000 nM dose, indicating PTSD diagnosis-specific responses to HCort. **b**, Significant *NGN2-*DRGs correctly classify PTSD(+) from PTSD(–) participants using an unsupervised approach. **c**, Meta-analysis of expression LogFC DRGs (differences observed between PTSD(+) and PTSD(–)) were plotted against –log(*P* value). Pink points indicate significantly DEGs in the meta-analysis, representing PTSD case-specific response genes to HCort. Sample-size-based meta-analysis was conducted using METAL with a Cochran’s *Q* test for heterogeneity. Benjamini–Hochberg adjusted *P* values are reported to correct for multiple testing. **d**, Gene set enrichment of significant DRGs across psychiatric disorder gene sets (epilepsy^69^, developmental delay^70^, ASD^71–76^, ID^77,78^, SCZ^79,80^ and FMRP targets^81^). **e**, The interactive effect of PTSD diagnosis and HCort exposure on gene expression are modeled, and three main observed patterns of direction of effect in significantly interactive genes are represented here. Interaction statistics were derived from the linear model with a diagnosis by HCort interaction coefficient. From among the 740 genes with significant Benjamini–Hochberg-adjusted *P* values <0.05, genes were identified that responded significantly to HCort in either cases or controls by performing one-way ANOVA on log_2_CPM normalized expression to increasing HCort exposure separately in PTSD cases and controls. We denote genes with a significant ANOVA *P* value in controls but not in PTSD cases as ‘PTSD hypo-responsive’, genes with a significant ANOVA *P* value in both PTSD cases and controls but with opposite directions of effect as ‘interactive’ and genes with a significant ANOVA *P* value to HCort in PTSD cases but not in controls as ‘PTSD hyper-responsive’. Data are presented as minimum, first quartile, median, third quartile and maximum. The patterns of three representative genes that indicate patterns of PTSD by HCort interaction, PTSD hyper-responsivity and PTSD hypo-responsivity are plotted, demonstrating examples of three biologically meaningful patterns of diagnosis by HCort interaction. **f**, logFC of all significantly interactive diagnosis by HCort genes are plotted against the *P* value of their interaction term, with most significant genes representing those with most significant interactive effects.

PTSD-specific transcriptional responses to glucocorticoid exposure were examined in human neurons. Baseline gene expression profiles (vehicle; 0 nM HCort) between PTSD(+) and PTSD(–) groups were compared, with no significant differences in gene expression observed (*Q* value < 0.05) (Fig. 4a). Linear contrast analysis was used to examine the extent to which genes respond differently to HCort, relative to baseline, in PTSD(+) relative to PTSD(–) combat veterans, here termed differential response genes (DRGs); 1,016 and 402 PTSD-specific DRGs were identified in *NGN2-*neurons following treatment with 100 nM and 1,000 nM HCort, respectively (Fig. 4a and Supplementary Table 6). The significant DRGs in *NGN2-*neurons predicted PTSD; for each individual, unsupervised classification revealed a clear pattern of HCort response dysregulation that correctly classified *NGN2-*neurons from PTSD(+) and PTSD(–) groups (Fig. 4b). Meta-analysis revealed shared DRGs across batches (Fig. 4c), demonstrating the validity of this PTSD signature. A gene set enrichment analysis of our DRGs against 17 psychiatric disorder gene sets revealed significant enrichment in PM PTSD dorsolateral prefrontal cortex (dlPFC)^27^ (*P* = 2.30 × 10^–7^*)*, and orbitofrontal cortex (OFC) (*P* = 0.016) as well as female and male specific interpersonal traumas (*P* = 1.03 × 10^–6^, *P* = 0.005, respectively) (Fig. 4d). Altogether, PTSD-specific neuronal DEGs are not detectable at baseline, are most significant in response to low (100 nM) glucocorticoid exposure and enriched for PM PTSD transcriptomic signatures.

**Fig. 5:**
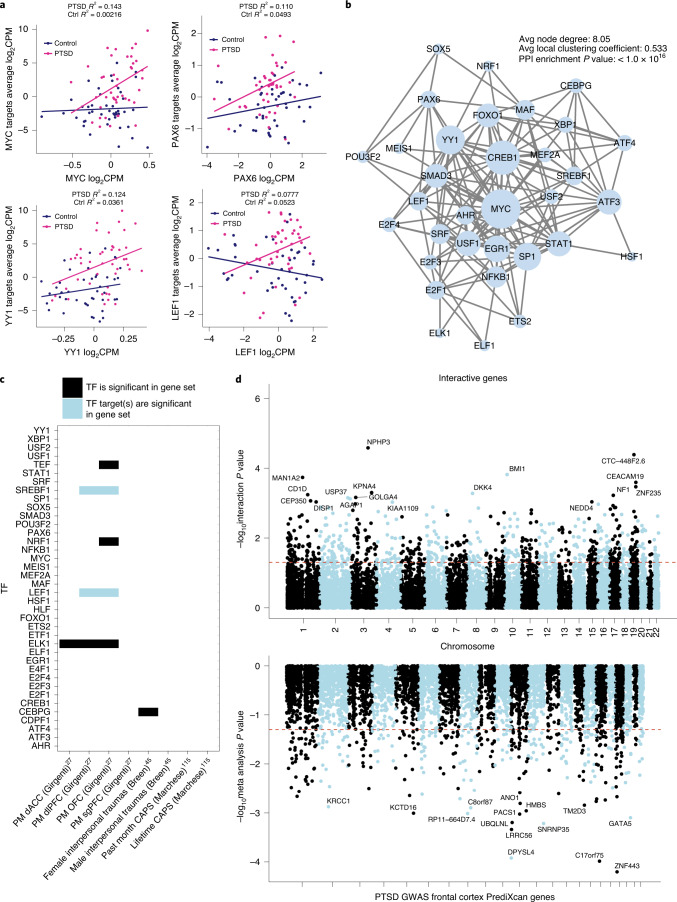
TFs driving PTSD hyper-responsivity. **a**, PTSD hyper-responsive genes were shown to be enriched for several TF targets. Four of the enriched TFs driving hyper-responsivity in PTSD cases are shown here. Normalized expression of the TF is graphed on the *x* axis with average expression of the TF targets on the *y* axis in PTSD case and control *NGN2*-neurons stimulated with HCort, demonstrating differential regulation in stimulated cases versus controls. **b**, Network visualization of PPIs amongst identified TFs mediating PTSD hyper-responsivity. STRING analysis was used to identify the observed number of edges. Enrichment of observed edges was assessed against expected edges to determine a PPI *P* value < 1.0 × 10–^16^. **c**, Overlap of TFs (black) and their targets (blue) identified in our study with significantly DEGs in other PTSD studies. dACC, dorsal anterior cingulate cortex; sgPFC, subgenual prefrontal cortex. **d**, Manhattan plot of significantly interactive genes in our study compared with Manhattan plot of imputed expression from PTSD GWAS indicating spatial orientation of significantly interactive genes.

The dose-dependent impact of HCort on PTSD case/control status was tested, revealing 740 genes with a significant diagnosis by HCort exposure interactive effect (FDR < 5%) (Fig. 4f). From this, three gene categories of interest were evaluated: (1) PTSD by HCort interaction, where expression effect with increasing HCort exposure was significant and in opposite directions in cases and controls (1 gene at FDR < 5%); (2) PTSD hypo-responsivity, where increasing HCort exposure only caused a significant expression change in controls, but not cases (84 genes at FDR < 5%) and (3) PTSD hyper-responsivity, where increasing HCort exposure caused a significant expression change in cases, but not controls (312 genes at FDR < 5%) (Fig. 4e). To assess spatial association of significantly interactive genes with common variants associated in PTSD, we mapped imputed expression of PTSD GWAS^3^ variants against the significance of the HCort by PTSD interaction term (Fig. 5d). Shared peaks were visible in chromosomes 10, 17 and 19.

### Transcription factor regulation of PTSD hyper-responsivity

Given previous reports of glucocorticoid hyper-responsivity in PTSD^6^, putative neuronal driver genes that may mediate this hyper-responsivity were predicted. The FUMA pipeline was used to perform gene set enrichment for transcription factor (TF) targets, finding 38 significantly enriched TFs for which our hyper-responsive genes were targets. To examine differences in regulatory function of these TFs within our study, the relationship between TF expression and target expression was investigated in PTSD cases and controls treated with HCort (Fig. 5a). In a subset of putative driver genes, increased TF levels more tightly corresponded to increased expression of target genes in neurons derived from PTSD cases than controls. These different patterns of TF coregulation in PTSD suggest a significant role for these TFs in driving PTSD-specific transcriptional response. The coregulation network was mapped by visualizing PPIs (Fig. 5b), finding a significant observed number of edges (*P* < 1.0 × 10^–16^), showing that linked expression of genes in this network translate to a shared effector regulation. Four of these 38 TFs, and two targets of identified TFs, were noted to be differentially expressed in previously reported PM^27^ and/or blood^47^ studies of PTSD (*P* value of 0.011, binomial test with an α of 0.05) (Fig. 5c). Altogether, coordinated transcriptional regulation and proteomic interaction of a network of TFs with shared effector regulation may mediate glucocorticoid hyper-responsiveness in PTSD.

## Discussion

Here, an hiPSC-based model was used to explore the cell-type-specific and diagnosis-dependent glucocorticoid treatment responses of PBMCs and *NGN2*-glutamatergic neurons derived from male combat-exposed PTSD cases (*n* = 19 hiPSC donors and *n* = 20 PBMC donors) and controls (*n* = 20 hiPSC donors and *n* = 20 PBMC donors). Both blood and neuronal glucocorticoid responses were significantly enriched for immune response genes; neuronal glucocorticoid responses were also associated with brain development and neurodevelopmental disorder genes. Although a PTSD diagnosis-specific signature was not detectable at baseline in either cell type, glucocorticoid hypersensitivity in PTSD was observed, with diagnosis-specific effects greatest in neurons at low HCort doses.

These findings are consistent with the glucocorticoid hypersensitivity hypothesis; for example, patients with PTSD have altered blood sensitivity to glucocorticoids^6^ and perturbations in glucocorticoid receptor signaling have been shown for PTSD in PM brain tissue^10^. That the PTSD-relevant gene set enrichment in HCort neuronal response was significant only if the effect of PTSD diagnosis was included in our model (compare Fig. 4d and Fig. 2e) suggests that environmental stress alone, independent of genetic risk, is insufficient to recapitulate PTSD biology. Further studies into the genetic risk elements imparting PTSD-specific HCort response, such as detecting HCort-responsive expression quantitative trait loci, may fine-map PTSD-associated risk loci. This will allow for more formal studies of colocalization, expanding on Fig. 5d, to spatially associate noncoding risk elements in PTSD with functional consequences. These findings emphasize the crucial role of stress response in PTSD risk but cannot rule out the potential impact of subsequent stress recovery; future work will be necessary to resolve the relative contributions of these alternatives.

The cell type(s) of origin in PTSD remain unresolved. It will be critical to explore diagnosis-dependent glucocorticoid responses across additional subtypes of brain cells to establish more physiologically relevant models. Although robust upregulation of immune response gene modules was observed in both cell types, the cell-type specific differences were confounded by cohort composition as well as experimental differences in the methods of glucocorticoid treatment used in each cell type (PBMCs: 72 h with 2.5 nM, 5 nM or 50 nM DEX; *NGN2*-neurons: 24 h with 100 nM, 1,000 nM or 2,500 nM HCort). Are blood-specific glucocorticoid changes a critical part of disease risk, initiation and/or progression, resulting in the release of additional cytokines and inflammatory molecules that impact brain function? Or are they simply an incomplete biomarker of causal events occurring in the brain? Within the brain, are neuron-specific responses adequate to interpret G×E interactions? Certainly, future experiments should investigate cell-autonomous and noncell-autonomous effects across a larger variety of neuronal, astroglial and microglial cell types. Moreover, an increasing amount of direct brain imaging^48–50^ and recent genomic analyses implicate specific subtypes of inhibitory GABAergic neurons in PTSD^27,51^. Transcriptome-wide association studies using PTSD GWAS^52^, as well as two independent transcriptomic studies of PM brain tissue from PTSD cases and controls^27,51^, associated GABAergic signaling with PTSD risk and illness state. This suggests that decreases in GABAergic activity confer susceptibility to traumatic stress and may be involved in the pathophysiology of PTSD^17^. Therefore, inclusion of GABAergic neurons, alone and in coculture with glutamatergic neurons, would be of particular interest in subsequent studies of neuronal glucocorticoid response.

Given the similarity of hiPSC-derived neurons to fetal brain cells^53–57^, our hiPSC-based model of PTSD may best reflect the G×E impact of developmental stress exposure, as demonstrated by robust enrichments for expression of autism spectrum disorder (ASD) risk genes, as observed here and previously^58^. Consistent with this, glucocorticoid treatment increases the proliferation of hiPSC-derived neural progenitor cells^59^, and impairs neuronal differentiation and maturation in hiPSC-derived neurons^20,60^ and primary mouse neurons^61^. Nonetheless, it would be an oversimplification to speculate that glucocorticoid response in hiPSC-derived neurons represents the ‘pretrauma state’, and that analyses of patient PBMCs and brain tissue reflect the ‘post-trauma state’. Stress impacts risk to psychiatric disorders throughout the lifespan—across prenatal development^62,63^, childhood^52^ and adulthood^52,64,65^. Moreover, the significant and positive relationship between our observed associations in *NGN2*-neurons and previously demonstrated transcriptional PTSD signatures in PM brains suggests that some impacts of glucocorticoid exposure may persist through to adulthood. Notably, glucocorticoid stimulation is not a specific model for PTSD; stress response is comorbid in many psychiatric disorders. HCort-responsive genes therefore likely represent aspects of stress response shared across PTSD and other neuropsychiatric disorders, such as the shared impact on social cognition between PTSD and ASD^66,67^. Combined analysis of context-dependent hiPSC models with cross-lifespan datasets, such as PM brains, may uncover long-term glucocorticoid-dependent PTSD signatures with which to refine hallmarks of PTSD susceptibility following combat exposure.

Because trauma exposure, even in the absence of PTSD, can impact glucocorticoid response^68^, future studies may usefully include donors with no trauma exposure. Toward this exploration, hCort response in *NGN2*-neurons was examined from one additional control without a reported trauma exposure, observing 13% overlap (805 of 6,190) of our HCort-responsive neuronal genes (exact binomial test 0.1300485). Likewise, glucocorticoid response in hiPSC-derived cerebral organoids from healthy and presumably trauma-free controls^20^ induced 16% (989 of 6,190) of the HCort-responsive genes here detected in *NGN2*-neurons (exact binomial test 0.1597738, *P* < 2.2 × 10^–16^).

hiPSC-derived neurons represent a promising platform with which to screen for genetic and/or pharmacological interventions that can prevent, ameliorate or reverse acute or chronic neuronal responses to stress. While glucocorticoid-responsive transcriptomic changes are sufficient to stratify trauma-exposed PTSD cases and controls, they are not yet clinically informative. Clinical utility is limited by variability of traumatic exposures, complexity of involved cell types, impracticality of individualized hiPSC culture and transcriptomic analysis, poor understanding of the specific genetic loci mediating differential HCort response, and lack of in-depth characterization of how in vitro phenotypes correspond to clinical phenotypes or treatment susceptibility. To translate our findings into clinically meaningful risk-stratification, it is imperative that future studies test whether our signatures relate to clinical severity and/or treatment responsiveness, and thereby uncover new therapeutic target(s) for PTSD. The current work indicates that if the biological mediators of environmental risk can be predicted, then hiPSC-based models can be used to test genotype-dependent and cell-type-specific environmental responses. Thus, given the critical importance of gene by stress (and G×E more broadly), we believe that, moving forward, functional genomics approaches must integrate stem cell models and genome engineering to resolve the impact of patient-specific variants across cell types and environmental stressors. Our hope is that dissecting how disease risk variants interact with the environment across the diverse cell types that comprise the human brain will ultimately improve diagnostics, predict clinical trajectories and identify pathways that could serve as presymptomatic targets of therapeutic intervention.

## Methods

### Participants

A total of 49 individuals were recruited to yield well-matched and largely overlapping (30 shared individuals) hiPSC and PBMC cohorts, comprised of combat veterans with (*n* = 19 hiPSC donors and *n* = 20 PBMC donors) and without (*n* = 20 hiPSC donors and *n* = 20 PBMC donors) PTSD. Detailed information about donor breakdown in the hiPSC and PBMC studies, by experimental batch, is presented in Supplementary Table 2. Participants in this cross-sectional study were combat-exposed Operation Enduring Freedom (OEF), Operation Iraqi Freedom (OIF) and Operation New Dawn (OND) veterans with and without PTSD who provided written informed consent (VA HS no. YEH-16-03 and ISMMS HS no. 15-00886) and from whom a viable blood and/or fibroblast sample was provided and sufficient RNA for genome-wide expression analyses was extracted. Participants were compensated $300 for involvement in this study. Eligibility for participation was determined as previously described^23^. Participants were included serially in the order in which they consented until the enrollment targets were attained. All participants reported a DSM-IV criterion A combat trauma^82^; all experienced deployment to active military combat zones and experienced one or more significant combat-related traumas. Individuals with and without PTSD did not have significant differences in childhood or predeployment trauma, deployment number or cumulative duration. Participants underwent psychological evaluation using the Structured Clinical Interview for DSM-5 (SCID)^83^ and the Clinician Administered PTSD Scale (CAPS)^84^ for determination of PTSD diagnosis and severity. Eligibility criteria and thresholds were based on CAPS for DSM-IV; PTSD(+) had a current CAPS-IV total score above 40 (frequency plus intensity), whereas PTSD(–) participants were combat-exposed veterans with a total score below 40. Although DSM-IV criteria for PTSD were used for inclusion, note that PTSD(+) participants also met criteria for PTSD based on DSM-5. Diagnostic and clinical exclusions included: (1) presence of moderate or severe substance use disorder within the past 6 months, (2) lifetime history of primary psychotic or bipolar I disorders (BD), (3) self-reported history of moderate or severe traumatic brain injury, (4) neurological disorder or main systemic illness, (5) treatment with systemic steroids and, for PTSD(−) only, (6) current or recurrent major depressive disorder (MDD). Psychotropic medication was permitted, but dosage stabilization for at least 2 weeks was required. Around 20% of individuals across both groups are currently treated with psychiatric medications. Current oral steroid treatment was an exclusion based on the impact of systemic steroids on the hypothalamic-pituitary-adrenal axis. Given the small sample size, there was no additional matching performed for clinical characteristics such as index trauma types, duration of the disorder, comorbidities and psychiatric medications at time of recruitment. Available clinical information is summarized in Supplementary Table 1 and presented in detail in Supplementary Table 2.

### Biopsy collection and generation/validation of hiPSCs

Biological samples (blood and fibroblast) were collected from eligible participants at the James J. Peters (JJP) Veterans Affairs Medical Center (VAMC). Blood processing occurred at the JJP VAMC and fibroblasts were transferred to the New York Stem Cell Foundation Research Institute (NYSCF). All hiPSCs were reprogrammed together in randomized batches as fibroblast biopsies were obtained over time.

Skin fibroblasts of PTSD and non-PTSD participants were collected via skin punch biopsy taken from the upper buttocks. Upon collection, biopsies were placed immediately in Biopsy Collection Medium (Cascade Biologics Medium 106 (Life Technologies, catalog no. M-106-500), 1× Antibiotic-Antimycotic (Life Technologies, catalog no. 15240-062) and LSGS (Life Technologies, catalog no. S-003-10)) and stored at 4 °C for a maximum of 24 h. Biopsies were dissected into pieces of around 1 mm^3^ and plated in Biopsy Plating Medium (Knockout-DMEM (Life Technologies, catalog no. 10829-018), 10% fetal bovine serum (Life Technologies, catalog no. 100821-147), 2 mM GlutaMAX (Life Technologies, catalog no. 35050-061), 0.1 mM MEM nonessential amino acids (Life Technologies, catalog no. 11140-050), 1× Antibiotic-Antimycotic (Life Technologies, catalog no. 15240-062) and 0.1 mM 2-mercaptoethanol (Life Technologies, catalog no. 21985-023)). Upon the outgrowth of fibroblasts, samples were entered into an automated pipeline producing vials of cells for both hiPSC reprogramming and backup stock. A cell pellet was collected, with DNA isolated using an EPMotion and the ReliaPrepTM 96 gDNA Miniprep HT System (Promega, catalog no. A2671). This DNA was used to confirm sample identity throughout the reprogramming process. Fibroblasts were reprogrammed using modified mRNA (Reprocell, catalog no. 000076) and enriched using antifibroblast meads (Miltentyi Biotec, catalog no. 130-050-601) in automated procedures. Reprogrammed hiPSCs were then expanded using PSC Feeder Free Medium (Thermo Fisher Scientific, catalog no. A14577SA) and grown on Cultrex-coated plates (R&D Systems, catalog no. 3434-010-02). Cells were routinely passaged using our automated platform in the presence of Accutase (Thermo Fisher, catalog no. A11105-01). Following passage, cells were maintained in PSC medium supplemented with 1 µM thiazovivin (Sigma-Aldrich, catalog no. SML1045-25MG). All cells were frozen in Synthafreeze (Thermo Fisher, catalog no. A12542-01) into master stocks.

As part of the hiPSC validation process, all samples were tested for the absence of Mycoplasma (Lonza, catalog no. LT07-710) and confirmed to be sterile (Hardy Diagnostics, catalog no. K82). Samples were confirmed to key karyotypically normal using the Illumina Core Exome Genotyping Chip (Illumina, catalog no. 20030770) and cnvPartition v.3.2.0 (Illumina, Genome Studio). No cell lines displayed karyotypic abnormalities (no reported copy number variations ⫺2.5 MB in size). All reported copy number variations are shown in each certificate of analysis. hiPSC lines were confirmed to be viable post-thaw, achieving a minimum of 50% confluency within 10 days). Sample identity testing was performed using the SNPTrace assay, confirming correct sample association between parental fibroblast and hiPSC line. Gene expression analysis was using a custom Nanostring panel^85^ to confirm expression of pluripotency markers such as *POU5F1*, *NANOG* and *SOX2*, and lack of expression of early differentiation markers such as *AFP* (Mesoderm), *SOX17* (Endoderm) and *NR2F2* (Ectoderm). A scorecard panel was used to confirm propensity to differentiate^85^. All hiPSC lines used in this study passed the above qualify control and have a certificate of analysis.

### PBMC isolation and culture

Isolation of PBMCs, cell culture and incubation with DEX were performed as described^23^ (Fig. 1a). PBMC data from *n* = 10 combat veterans with PTSD and *n* = 10 without PTSD is first reported here (batch A) and *n* = 10 combat veterans with PTSD and *n* = 10 without PTSD was reported previously^23^ (batch B) (Supplementary Table 2). In brief, blood was collected in EDTA-containing vacutainer tubes (VWR) and PBMCs were isolated by density gradient centrifugation using Ficoll-Paque (GE Healthcare) and washed twice in HBSS (Thermo Fisher, 14175). Mononuclear cells were then counted manually using a Cellometer disposable counting chamber (Nexcelom Bioscience LLC). The cells were resuspended in complete RPMI, containing RPMI-1640 (Gibco), 10% fetal bovine serum, 50 U ml^–1^ penicillin–streptomycin mixture (Gibco) at a density of 1.75–2.00 × 10^6^ cells ml^–1^ of the medium. PBMCs were prepared at 2.5 × 10^6^ cells ml^–1^ in complete RPMI for DEX treatment experiments. Following incubation at 37 °C, 5% (v/v) CO_2_ for 72 h, the plates were centrifuged at 900*g* for 15 min at 4 °C and supernatant was collected and pooled from each DEX concentration well. The cell pellet on the bottom of each well was resuspended in TRIzol reagent. Cell lysates for each DEX dose were pooled, aliquoted and stored at −80 °C until RNA isolation.

### Automated generation of hiPSC-derived *NGN2*-neurons

Glutamatergic *NGN2*-neurons were generated from hiPSCs, using high-throughput automated differentiations, in two batches (batch 1 *n* = 15 versus 15; batch 2 *n* = 4 versus 5) (Supplementary Table 2), as previously described with some modifications^36^. hiPSCs were single cell passaged after a 20-min dissociation with Accutase (STEMCELL Technologies) at 37 °C and 5% CO_2_. A total of 1 million cells per well were plated in 12-well Cultrex-coated (R&D Systems, catalog no. 3434-010-02) tissue culture plates (Corning Costar) in PSC Feeder Free Medium (Thermo Fisher Scientific, catalog no. A14577SA) with 1 µM thiazovivin (Sigma-Aldrich, catalog no. SML1045). Lentivirus (generated by ALSTEM) carrying pLV-TetO-hNGN2-eGFP-Puro (Addgene, catalog no. 79823) and FUdeltaGW-rtTA (Addgene, catalog no. 19780) was diluted to a multiplicity of infection of one each (1 million genome counts of each vector per transduction) in 100 µl DPBS, no calcium, no magnesium (Thermo Fisher Scientific) and added directly after cell seeding. After 24 h, the medium was exchanged with Neural Induction Medium (NIM) comprising a 50:50 mix of DMEM/F12 and Neurobasal, with 1× B27 plus vitamin A, 1× N2, 1× Glutamax (Thermo Fisher Scientific) and 1 µM doxycycline hyclate (Sigma-Aldrich). After 24 h, the medium was removed and NIM was added with doxycycline plus 5 µg ml^–1^ puromycin. A full medium exchange was performed the next day with NIM plus doxycycline and puromycin; 24 h later, cells were passaged by incubating with Accutase for 30 min at 37 °C and 5% CO_2_. A series of 96-well plates (PerkinElmer CellCarrier Ultra) were coated with 0.1% polyethylenimine (Sigma-Aldrich, catalog no. 408727) in 0.1 M borate buffer pH 8.4 for 30 min at room temperature, washed five times with water and prefilled with 100 µl per well of neural coating medium comprising Brainphys medium (STEMCELL Technologies) with 1× B27 plus vitamin A, 1 µM thiazovivin, 5 µg ml^–1^ puromycin, 250 µM dibutyryl cAMP (dbcAMP, Sigma-Aldrich), 40 ng ml^–1^ brain-derived neurotrophic factor (BDNF, R&D Systems), 40 ng ml^–1^ glial cell line-derived neurotrophic factor (GDNF, R&D Systems), 200 µM ascorbic acid (Sigma-Aldrich) and 10 µg ml^–1^ natural mouse laminin (Sigma-Aldrich). A sample of cells were stained with 10 µg ml^–1^ Hoechst plus 1:500 acridine orange/propidium iodide solution and counted on a Celigo imager (Nexcelom Bioscience). A total of 50,000 cells per well were seeded into neural coating medium-filled 96-well plates in 100 µl per well of neural medium comprising Brainphys medium with 1× B27 plus vitamin A, 1 µM thiazovivin, 5 µg ml^–1^ puromycin, 250 µM dbcAMP, 40 ng ml^–1^ BDNF, 40 ng ml^–1^ GDNF, 200 µM ascorbic acid and 1 µg ml^–1^ natural mouse laminin. At 24 h after seeding, medium was exchanged for neural selection medium comprising Brainphys medium with 1× B27 plus vitamin A, 250 µM dbcAMP, 40 ng ml^–1^ BDNF, 40 ng ml^–1^ GDNF, 200 µM ascorbic acid, 1 µg ml^–1^ natural mouse laminin and 2 µM arabinosylcytosine (Ara-C, Sigma-Aldrich). After 48 h, the neural selection medium was fully exchanged and after a further 48 h the medium was fully exchanged with neural maintenance medium (NMM) comprising Brainphys medium with 1× B27 plus vitamin A, 250 µM dbcAMP, 40 ng ml^–1^ BDNF, 40 ng ml^–1^ GDNF, 200 µM ascorbic acid and 1 µg ml^–1^ natural mouse laminin. Thereafter, every 48 h, half the medium was exchanged with NMM until day 21 posttransduction passage. In Batch 1, all medium exchanges were performed using a Hamilton Star liquid handler set to 5 µl s^–1^ for aspirate and dispense as part of the NYSCF Global Stem Cell Array® Team^86^. Passages were fully automated and performed on a robotic cluster comprising a Thermo Fisher Scientific C24 Cytomat incubator, a Hamilton Star liquid handler, an Agilent microplate centrifuge, a Precise Automation PreciseFlex 400 Sample Handler and a PerkinElmer Opera Phenix. In Batch 2, although medium exchanges were fully automated, passages were performed manually.

At harvest, medium was removed using the Bluewasher (BlueCatBio) and cells were lysed for 5 min using RLT plus buffer (Qiagen), snap frozen on dry ice and stored at −80 °C. A replicate plate was fixed for immunofluorescence analysis by adding 32% paraformaldehyde (Electron Microscopy Sciences) directly to medium to a final concentration of 4% and incubated at room temp for 15 min. Cells were washed three times with HBSS (Thermo Fisher Scientific), stained overnight with mouse anti-Nestin 1:3,000 (Millipore, catalog no. 09-0024), chicken anti-MAP2 1:3,000 (Abcam, catalog no. 09-0006) in 5% normal goat serum (Jackson ImmunoResearch) in 0.1% Triton X-100 (Thermo Fisher Scientific) in HBSS. Primary antibodies were counterstained with goat anti-mouse Alexa Fluor 555 and goat anti-chicken Alexa Fluor 647 and 10 µg ml^–1^ Hoechst for 1 h at room temp. Cells were washed three times with HBSS. Batch 1: nine fields (×40 magnification) were imaged per well (one well per condition per line) using the PerkinElmer Opera Phenix microscope. Batch 2: nine fields (×20 magnification) were imaged per well (two wells per hiPSC line) using the ArrayScan automated microscope (Thermo Fisher Scientific). Interwell variability in neuronal identity and maturity was assessed using automated image analyses. NESTIN-positive neural progenitor cells were demarcated from MAP2-positive postmitotic neurons (Extended Data Fig. 1). Variation between batches may reflect discrepancies in imaging methods, rather than biological differences in neuronal morphology or maturity.

### Glucocorticoid treatment

Preliminary studies were conducted to identify optimal culture and glucocorticoid stimulation conditions. These pilot studies sought to evaluate the transcriptional effects of HCort and DEX on *NGN2*-neurons, and optimize the length of glucocorticoid treatment and concentrations. Neither quantitative PCR for six glucocorticoid regulatory genes (covering ten concentrations of DEX) nor RNA-seq (covering three concentrations of DEX) revealed significant gene expression differences following 72 h of exposure^87^, consistent with minimal upregulation of *FKBP5* mRNA expression following DEX treatment of hiPSC neurons^19^. This is consistent with previous reports of DEX treatment of primary cultures, which found neurons to be significantly less responsive than astrocytes^88^. Together, our preliminary data uphold the view that astrocyte^88^ or endothelial^89^ response, rather than neuronal response, mediates brain-level effects of DEX treatment. As our preliminary experiments found a strong genome-wide effect of HCort treatment, we used HCort treatment for neuronal glucocorticoid exposure. Serum measurements of cortisol in patients with and without PTSD have been found to average around 492.52 nM^90^, intermediate between our 100 and 1,000 nM doses. HCort treatment medium was prepared by first dissolving HCort (Sigma-Aldrich, catalog no. H0888) in ethanol to make a 2.8 mM stock. HCort ethanol stock was then diluted to 0.2 mM in HBSS. Ethanol was equalized to 15 µM in control and all treatment media. DEX treatment medium was prepared by dissolving DEX (Sigma-Aldrich, catalog no. D1156) in HBSS^6^. The final treatment medium was prepared by diluting HCort or DEX stocks into NMM/RPMI, before applying to cells by fully exchanging medium. Neurons were treated with HCort for 24 h (baseline, 100 nM, 1,000 nM and 2,500 nM), PBMCs with DEX for 72 h (baseline, 2.5 nM, 5 nM and 50 nM). Baseline medium conditions are estimated to contain 58 nM of corticosterone from neuronal supplement B27 (Thermo Fisher), which may predispose neurons to a higher effective concentration for glucocorticoid stimulation^91^, and may bias responsive genes toward those that respond to severe stressors, rather than homeostatic or regulatory changes, such as circadian rhythms.

### RNA extraction and quality control

For *NGN2-*neurons, RNA was harvested with an RNeasy Plus Micro Kit (Qiagen). For PBMCs, RNA was extracted from TRIzol-lysed PBMCs using the miRNAeasy Mini Kit (Qiagen). Following each extraction, RNA quantity was measured using a Nano Drop 2000 Spectrophotometer (Thermo Scientific) and RNA quality and integrity was measured with an Agilent 2100 Bioanalyzer (Agilent). All RNA integrity numbers (RINs) in the current study were high: *NGN2-*neurons (8.8 ± 0.53) and PBMCs (7.5 ± 0.95).

### RNA-seq data generation

A low-input RNA-seq protocol was applied for the generation of RNA-seq data from *NGN2*-neurons. Specifically, polyA enriched RNAs were subjected to RNA-seq library preparation using the SMART-Seq v.4 Kit (SSv.4; Takara) and sequenced using a paired-end 150 bp configuration with 30 M supporting reads per sample. For RNA-seq data generation from PBMCs, Ribo-zero rRNA deleted RNAs were subjected to RNA-sequencing library preparation using the Illumina TruSeq Stranded Total RNA kit (Illumina) and sequenced using a paired-end 150 bp configuration with 20 M supporting reads per sample.

### RNA-seq data preprocessing

All RNA-seq FASTQ files underwent matching analytical procedures, as described previously. In brief, resulting short reads with Illumina adapters were trimmed and low-quality reads were filtered using TrimGalore^92^ (*–illumina* option). All high-quality reads were then processed for alignment using the hg38 reference and the ultrafast universal RNA-seq aligner STAR with default parameters^93^. Mapped bam files were sorted using Samtools and short read data were quantified using featureCounts^94^ with the following parameters: -T 5, -t exon and -g gene_id. Subsequently, all read counts were exported and all downstream analyses were performed in the R statistical computing environment. Raw count data was subjected to nonspecific filtering to remove low-expressed genes that did not meet the requirement of a minimum of two counts per million (CPM) in at least around 40% of samples. This filtering threshold was applied to *NGN2*-neurons and PBMCs separately. All expression values were converted to log_2_ reads per kilobase of transcript, per million mapped reads (RPKM) and subjected to unsupervised principal component analysis (PCA) to identify and remove outlier samples that lay outside 95% confidence intervals from the grand averages. This identified two outlier samples in *NGN2*-neuron Batch 1 and one outlier sample in Batch 2 that were excluded from our analysis.

### Developmental specificity analysis

RNA-seq datasets from existing PM brain tissue and hiPSC models were integrated to validate the developmental specificity of our samples using a previously described approach^40^. In brief, a total of 16 independent studies were collected covering 2,716 independent samples and 12,140 genes. These samples cover a broad range of hiPSCs, neural progenitor cells, mature neurons, prenatal and postnatal brain tissues. All expression values were converted to log_2_ RPKM and collectively normalized using quantile normalization using the *limma* R package. Subsequently, for each independent sample present in our hiPSC neuron dataset, we performed pairwise correlation analysis (using Pearson’s correlation coefficients) across all independent samples and subsequently aggregated the correlation coefficients for each external study and/or cell type. Next, and as a complementary approach, all datasets were jointly analyzed and integrated using PCA (Extended Data Fig. 3).

### Differential gene expression analyses

**Extended Data Fig. 2:**
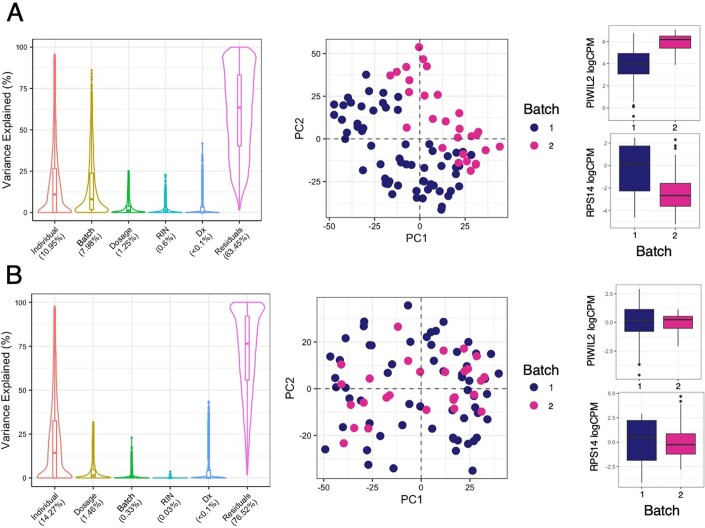
Adjustment of Batch effects. **a**, VariancePartition and PCA analysis on uncorrected data from two batches (batch 1, n = 30, batch 2, n = 9 biologically independent individuals) indicating large batch effect. Boxplot data presented are minimum, 1st quartile, median, 3rd quartile and maximum. Example of gene with large batch effect across batch 1 (n = 30) and batch 2 (n = 9). **b**, VariancePartition, PCA, and example gene after batch correction for same batches as above.

**Extended Data Fig. 3:**
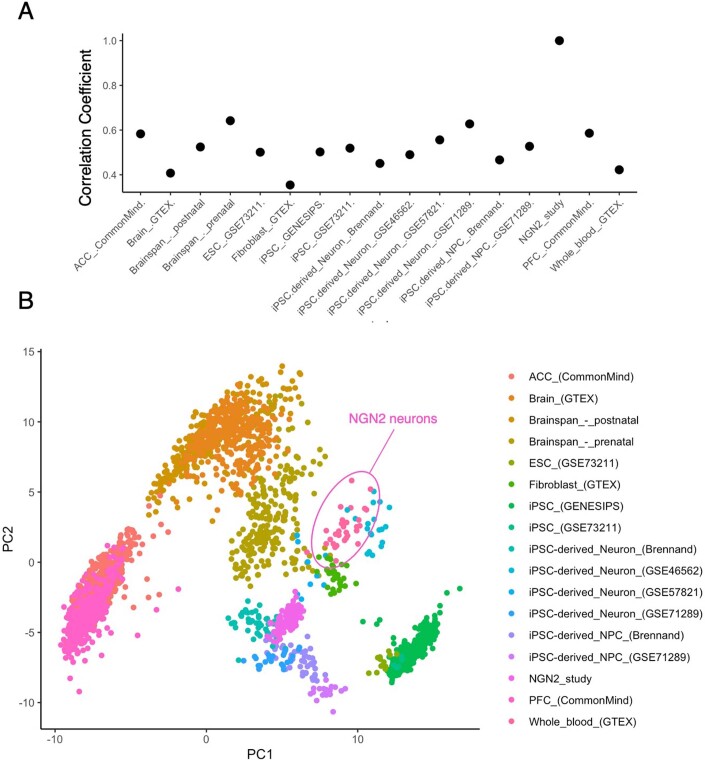
Developmental specificity analysis. **a**, Pairwise correlation between NGN2s from our study with cell types across 16 independent studies. **b**, PCA analysis of cell types within all 16 studies with our NGN2-neurons.

**Extended Data Fig. 4:**
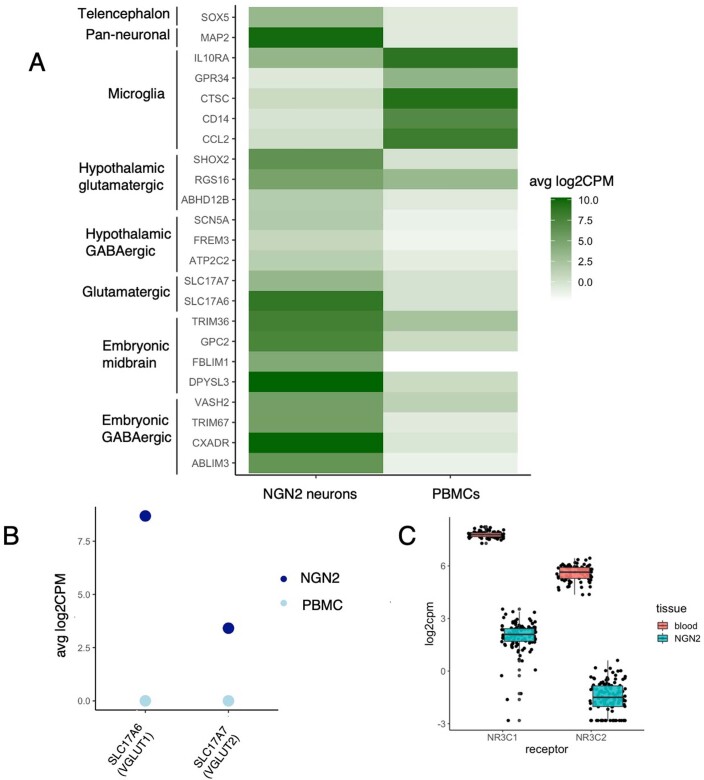
Neuronal fate specificity analysis. **a**, Expression of hallmark pan-neuronal and neuronal subtype specific genes in NGN2-neurons and PBMCs. **b**, Average log2CPM expression of VGLUT1 and VGLUT2 in NGN2-neurons and PBMCs. **c**, Expression of GR and MR in NGN2-neurons (from n = 39 individuals) and PBMCs (from n = 40 individuals). Boxplot data are presented as minimum, 1st quartile, median, 3rd quartile and maximum.

**Extended Data Fig. 5:**
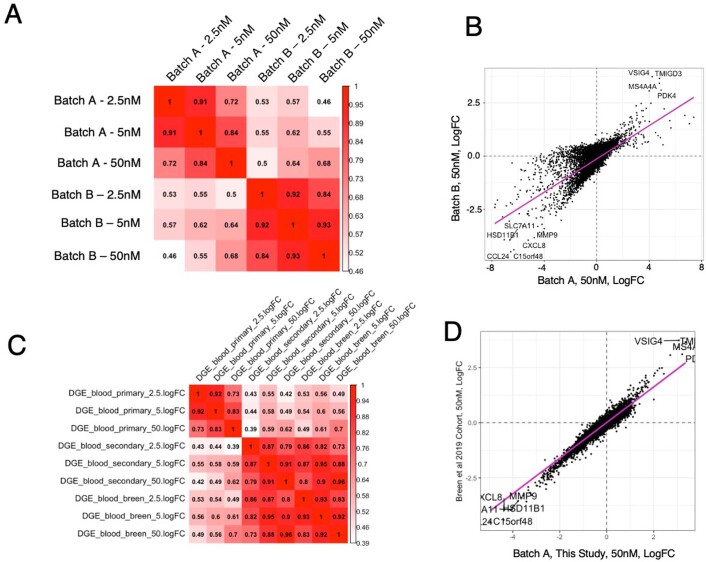
Comparison of PBMC batches. **a**, Pairwise correlations between PBMC batches. **b**, Transcriptome-wide correlation between batches at 50 nM DEX dose. **c**, Pairwise correlations between PBMC batches and Breen et al^23^. **d**, Transcriptome-wide correlation between our study and Breen et al.^23^ at the 50 nM DEX dose.

Transcriptional signatures were generated using a strategy adapted from Hoffman et al.^40^, using scripts available at www.synapse.org/hiPSC_COS. Gene expression values were normalized using VOOM^95^. Confounders explaining a significant proportion of variance in gene expression were identified using variancePartition^96^ (that is, experimental batch, treatment, individual as a repeated measure (that is, interdonor effects), PTSD diagnosis and RIN) (Extended Data Fig. 2a). A significant batch effect was observed (Extended Data Fig. 2a–c), which was corrected for by constructing a linear model of batch and extracting the residuals (Extended Data Fig. 2d–f). Next, differential gene expression analyses were conducted using a moderated *t* test from the R package *limma*^95^. Models examining the HCort-dependent, PTSD-independent effect (Figs. 1–3) included adjustment for the possible confounding influence of PSTD diagnosis and RIN, while PTSD-dependent models (Figs. 4 and 5) included diagnosis as a main outcome. Due to the repeated measures study design, where individuals are represented by multiple independent technical replicates, the duplicateCorrelation function was applied in the *limma* analysis and gene level significance values were adjusted for multiple testing using the Benjamini and Hochberg method to control the FDR. Genes with FDR < 5% were considered significantly differentially expressed. To integrate differential gene expression results between batches, summary statistics from differential gene expression analyses to both glucocorticoid-dependent and PTSD-dependent responses in each batch were meta-analyzed using METAL^97^. Significant results across both studies were identified using a Benjamini–Hochberg-adjusted *P* value threshold (FDR < 0.05).

### Neurite outgrowth analysis

To determine functional and mechanistic consequences of HCort treatment, *NGN2*-neurons were seeded as 1.5 × 10^4^ cells per well in a 96-well plate coated with 4× Matrigel at day 5. At day 6, *NGN2*-neurons were treated with HCort for 24 h (0 nM (vehicle), 100 nM, 1,000 nM and 2,500 nM) as in previous experiments. At day 7, cultures were fixed using 4% formaldehyde/sucrose in PBS with Ca^2+^ and Mg^2+^ for 10 min at room temperature (RT). Fixed cultures were washed twice in PBS and permeabilized and blocked using 0.1% Triton/2% normal donkey serum (NDS) in PBS for 2 h. Cultures were then incubated with primary antibody solution (1:1,000 MAP2 anti-chicken (Abcam, catalog no. ab5392) in PBS with 2% NDS) overnight at 4 °C. Cultures were then washed three times with PBS and incubated with secondary antibody solution (1:500 donkey anti-chicken Alexa 647 (Life technologies, catalog no. A10042) in PBS with 2% NDS) for 1 h at RT. Cultures were washed a further three times with PBS with the second wash containing 1 μg ml^–1^ 4,6-diamidino-2-phenylindole. Fixed cultures were then imaged on a CellInsight CX7 HCS Platform with a ×20 objective (0.4 numerical aperture) and neurite tracing analysis performed using the neurite tracing module in the Thermo Scientific HCS Studio v.4.0 Cell Analysis Software. A total of 12–24 wells were imaged per condition across a minimum of two independent cell lines, with nine images acquired per well for neurite tracing analysis. A Kruskal–Wallis test with a post hoc Dunn’s multiple comparisons test was performed on data for neurite length per neuron using Graphpad Prism. This analysis was performed in day 7 neurons to ensure optimal density to observe neurite outgrowth phenotypes, which are less quantifiable by these methods in more mature neurons.

### Weighted gene coexpression network analysis and functional annotation

**Extended Data Fig. 6:**
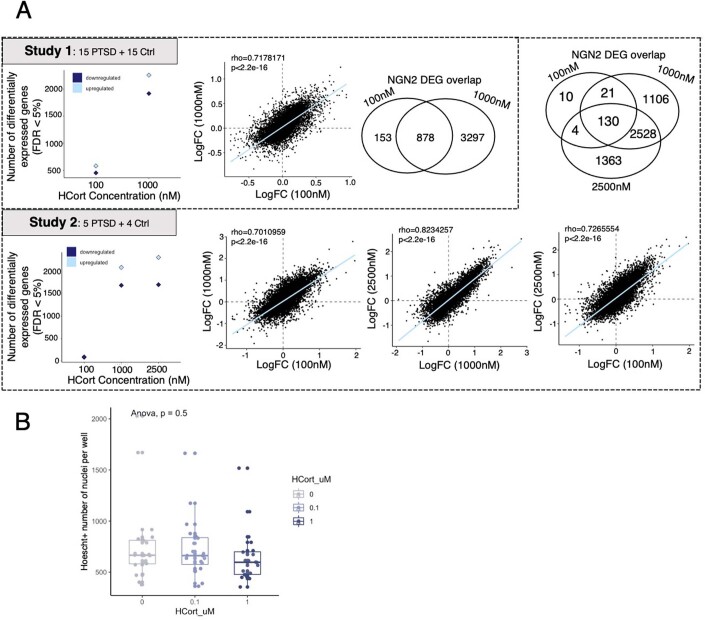
Comparison of NGN2 batches. **a**, Hcort-responsive DEGs across independent batches. Transcriptome-wide concordance is plotted between dosages for each batch. **b**, Quantification of cell number with HCort treatment showing no significant cell density between doses. One-way ANOVA was performed in n = 39 biologically independent individuals treated with each dose. Data are presented as minimum, 1st quartile, median, 3rd quartile and maximum.

**Extended Data Fig. 7:**
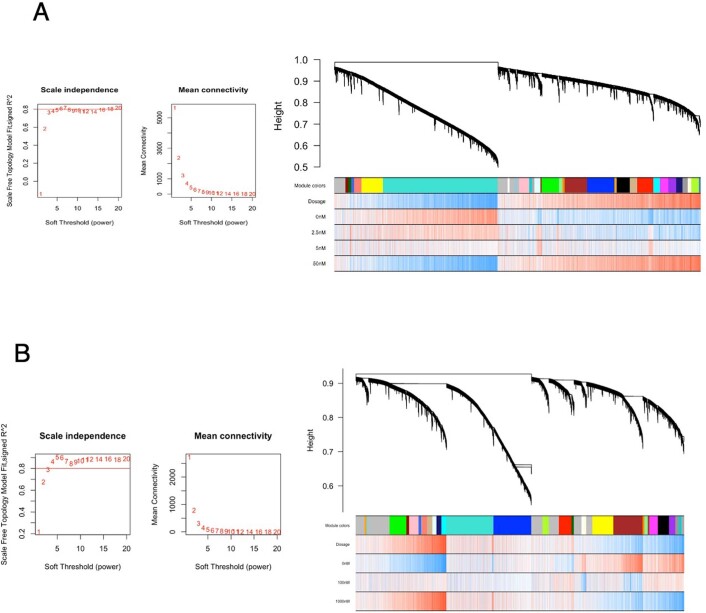
Weighted gene co-expression network analysis of (A) PBMCs and (B) NGN2-neurons. The β-power required to satisfy scale-free topology (SFT) and corresponding mean connectivity for gene co-expression network construction. Hierarchical gene cluster tree and module structure and gene-treatment color bands. The first color band underneath the tree indicates the detected modules and subsequent bands indicate treatment correlation, when red indicates a strong relationship and blue indicates a strong negative relationship.

**Extended Data Fig. 8:**
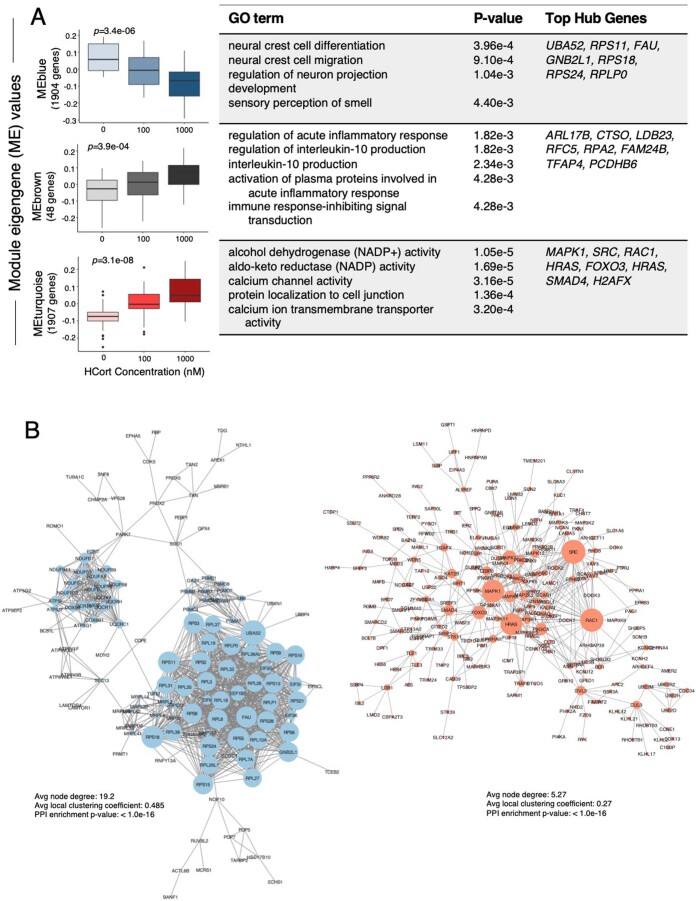
Unsigned WGCNA Network. **a**, Unsigned Weighted gene co-expression network analysis (WGCNA) identified coregulated gene modules. Pearson correlation was used to assess changes in module eigengene (ME) values with increasing concentration of HCort (p-values are labeled above each boxplot). Correlation p-values were derived from a two-tailed t-distribution. Data are presented as minimum, 1st quartile, median, 3rd quartile and maximum. Each module was subjected to gene ontology enrichment analysis and the topmost significant enrichment terms and their associated Benjamini–Hochberg adjusted P-values are displayed. Top hub genes (kME > 0.6) within each module are displayed for quick interpretation of GR-stimulated gene co-expression modules and candidate individual genes. **b**, Network visualization of protein-protein interactions within modules indicating clusters and network hubs. STRING analysis was used to identify the observed number of edges. Enrichment of observed edges was assessed against expected edges to determine a Protein Protein Interaction (PPI) p-value of p < 1.0e-16 for each network.

**Extended Data Fig. 9:**
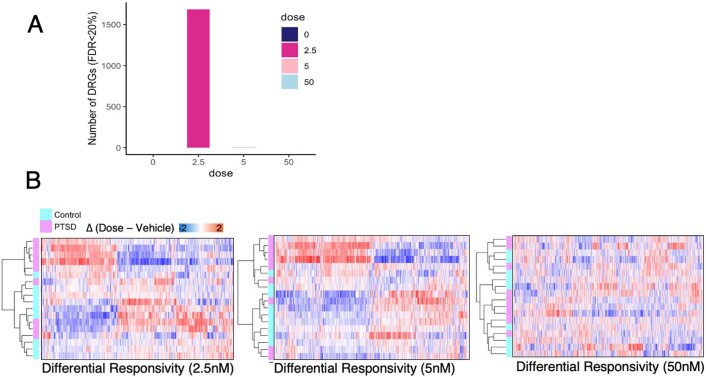
PTSD(+) specific responses to DEX in PBMCs. **a**, Genes that differ in their response to DEX in PTSD(+) donors compared to PTSD(-) donors, here termed differential response genes (DRGs), at an FDR threshold of 20% (nonsignificant) **b**, Unsupervised clustering of nominally significant PTSD DRGs.

Signed coexpression networks were built for PBMCs and *NGN2*-neurons using WGCNA^98^. To construct a global weighted network for each cell type, a total of 20,101 and 16,146 postquality-control genes were used in PBMCs and *NGN2*-neurons respectively. The absolute values of Biweight midcorrelation coefficients (optimal for small sample sizes) were calculated for all possible gene pairs within each cell type, and resulting values were transformed using a β-power (β = 4 for *NGN2-*neurons and β = 7 for PBMCs) so that the final correlation matrices followed an approximate scale-free topology (Extended Data Fig. 6). To determine which modules, and corresponding biological processes, were most associated with HCort/DEX, singular value decomposition of each module’s expression matrix was used; the resulting ME), equivalent to the first principal component, represented the overall expression profiles for each module. Each module was enriched for GO biological processes, molecular factors, cellular components and molecular pathways using ToppGene^99^. All genes passing nonspecific filtering in the current dataset were used as a genomic background. Only gene sets that passed a multiple test adjustment using the Benjamini–Hochberg procedure (adj. *P* < 0.05) were deemed significant. ME values were correlated with dosage by Pearson correlation. Protein–protein association networks were constructed using STRING^100^. For protein–protein association analysis of WGCNA modules, genes with high module membership (>0.8) were selected for STRING analysis and computation of PPI enrichment *P* values. Networks were visualized by cytoscape^101^.

### Gene coexpression module preservation analysis

Gene coexpression modules that were disrupted or created in response to glucocorticoids across *NGN2-*neurons and PBMCs, a permutation-based preservation statistic (Z_summary_)^2^ with 200 random permutations was used to measure the (dis)similarity in correlation patterns for the genes within these gene sets: Z_summary_ > 10 indicates strong evidence of preservation, 2 < Z_summary_ < 10 indicates weak-to-moderate evidence of preservation and Z_summary_ < 2 indicates minimal-to-no evidence of preservation. For this analysis, we specifically focused on dynamically regulated, glucocorticoid-responsive functional modules that were identified in either *NGN2-*neurons or PBMCs, respectively.

### Integration of disease-associated genes and gene sets

**Extended Data Fig. 10:**
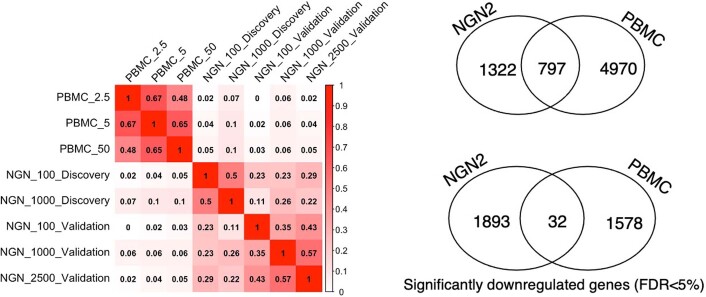
Concordance of PBMC signature with NGN2 signature. Pairwise Pearson-correlations between transcriptome-wide signatures of PBMC and NGN2 batches.

Concordance of observed PBMC and neuron transcriptomic signatures to glucocorticoid stimulation (Figs. 1e and 2e), or between PTSD cases and controls (Fig. 4d) and previously reported psychiatric disorder and neurodevelopmental expression patterns^102–108^ was determined (Extended Data Fig. 10). Psychiatric disorder enrichment was determined using genetic and genomic disease-related gene lists for PTSD, MDD, schizophrenia (SCZ), BD, ASD, alcohol use disorder, and inflammatory bowel disease (IBD)^27^. Glucocorticoid dysregulation of neurodevelopmental genes was examined using risk genes associated with epilepsy^69^, developmental delay^70^, autism spectrum disorder^71–76^, intellectual disability (ID)^77,78^, SCZ^79,80^ and fragile X mental retardation protein (FMRP) target^81^ genes. All gene sets were assessed by overrepresentation analysis^109^ using WebGestaltR^110^, with significant enrichment calculated using hypergeometric distribution. All *P* values from all gene sets were adjusted for multiple testing using the Benjamini–Hochberg procedure, using a *P* value < 0.05 threshold to determine significance. These gene lists are available in Supplementary Table 3.

### Clustering of PTSD expression patterns

To assess whether significant gene expression patterns could predict PTSD, the differential log_2_CPM of 1,016 and 402 PTSD-specific DRGs in *NGN2-*neurons following 100 nM and 1,000 nM of HCort, respectively, were plotted using pheatmap^111^. K-means clustering was applied with Euclidian distance using average linkage clustering and tested for observed clustering of PTSD(+) and PTSD(–) gene signatures (Fig. 4b).

### Interaction analysis

To test the interaction of HCort concentration with PTSD diagnosis, the linear effect of the HCort by PTSD interaction term was modeled using *limma*, adjusting for RIN and donor as a repeated measure. Of the 740 genes with significant Benjamini–Hochberg-adjusted *P* values <0.05, genes were identified that responded significantly to HCort in either cases or controls by performing one-way analysis of variance (ANOVA) on log_2_CPM normalized expression to increasing HCort exposure separately in PTSD cases and controls. Genes with a significant ANOVA *P* value in controls but not in PTSD cases as ‘PTSD hypo-responsive’, genes with a significant ANOVA *P* value in both PTSD cases and controls, but with opposite directions of effect as ‘interactive’ and genes with a significant ANOVA *P* value to HCort in PTSD cases but not in controls were labeled as ‘PTSD hyper-responsive’ (Fig. 4e). We selected these three categories to maximize biological interpretability, and due to previous reports of glucocorticoid hypersensitivity in PTSD, suggesting hyper- and hypo-sensitivity of targets as physiologically relevant to PTSD. To assess spatial association of genes with the most significant interactive effects with known common variant effectors involved in PTSD, expression of PTSD GWAS^3^ variants was imputed using PrediXcan^112^ and mapped against the significance of the HCort by PTSD interaction term in our study (Fig. 5d).

To identify upstream drivers of the PTSD-specific HCort response signature, enrichment for TF targets, defined based on MSigDB C3 gene set groupings^113^, was performed using the FUMA pipeline^114^. TFs were tested for their overlap with genes previously reported in PM brains^27^ of individuals with PTSD and genes associated with CAPS scores in whole blood^115^. The significance of the number of TFs enriched in PTSD gene sets was tested using a binomial test.

### Statistics and reproducibility

Sample sizes for this study were chosen based on comparisons with other idiopathic designs^40,116,117^. Using an isogenic within-donor design, we were adequately powered to resolve Hcort specific effects. No statistical method was used to predetermine sample size for case–control comparisons. Data was assumed to be normal due to log_2_-transformation of all expression data. RNA-seq sample data was excluded based on PCA of genome-wide log_2_RPKM values to identify outlier samples that lay outside 95% confidence intervals from the grand averages. This identified two outlier samples in *NGN2*-neuron Batch 1 and one outlier sample in Batch 2 that were excluded from our analysis. All experimentation and quality control on hiPSC lines was performed with blinding to disorder status. Sorting of samples occurred with HCort treatment conditions laid out with increasing dosages from top to bottom for both sample groups. Control and case samples were randomized across plates. RNA-seq from samples were randomized for sequencing.

### Reporting summary

Further information on research design is available in the Nature Research Reporting Summary linked to this article.

## Online content

Any methods, additional references, Nature Research reporting summaries, source data, extended data, supplementary information, acknowledgements, peer review information; details of author contributions and competing interests; and statements of data and code availability are available at 10.1038/s41593-022-01161-y.

## Supplementary information

Reporting Summary

Supplementary Tables Supplementary Table 1. Comparison of clinical characteristics of veterans with and without PTSD. Supplementary Table 2. a, Demographic information and PTSD severity of individuals included within this dataset. b, Data dictionary of terms used in demographic feature table. Supplementary Table 3. Curated disorder-associated gene sets. Supplementary Table 4. Results from meta-analysis of differential expression by batch for PBMCs and *NGN2*-neurons treated with HCort.-based meta-analysis was conducted using METAL with a Cochran’s Q test for heterogeneity. a, Meta-analysis results for PBMCs treated with 2.5 nM DEX. b, Meta-analysis results for PBMCs treated with 5 nM DEX. c, Meta-analysis results for PBMCs treated with 50 nM DEX. d, Meta-analysis results for *NGN2*-neurons treated with 100 nM HCort. e, Meta-analysis results for *NGN2*-neurons treated with 1,000 nM HCort. Supplementary Table 5. a,b, GO enrichment results of coregulated expression modules that vary significantly in PBMCs treated with DEX (a), and *NGN2*-neurons treated with HCort (b). Supplementary Table 6. a,b, Results from meta-analysis of PTSD-effect differential expression by batch for *NGN2*-neurons treated with 100 nM (a) and 1,000 nM of HCort (b). Sample size-based meta-analysis was conducted using METAL with a Cochran’s *Q*-test for heterogeneity.

## Data Availability

Owing to constraints reflecting the original patient consents, the hiPSCs will be made available by the authors upon reasonable request and IRB approval. RNA-seq files will be uploaded to the gene expression omnibus and will release following manuscript publication.

## Extended data

are available for this paper at 10.1038/s41593-022-01161-y.

## Code availability

All computational code is available at GitHub: https://github.com/BreenMS.

## References

[1] Yehuda, R. et al. Post-traumatic stress disorder. *Nat. Rev. Dis. Primers* **1**, 15057 (2015). 27189040 10.1038/nrdp.2015.57

[2] Kremen, W. S., Koenen, K. C., Afari, N. & Lyons, M. J. Twin studies of posttraumatic stress disorder: differentiating vulnerability factors from sequelae. *Neuropharmacology* **62**, 647–653 (2012). 21443892 10.1016/j.neuropharm.2011.03.012 PMC3153636

[3] Nievergelt, C. M. et al. International meta-analysis of PTSD genome-wide association studies identifies sex- and ancestry-specific genetic risk loci. *Nat. Commun.* **10**, 4558 (2019). 31594949 10.1038/s41467-019-12576-w PMC6783435

[4] Duncan, L. E. et al. Largest GWAS of PTSD (*N*=20 070) yields genetic overlap with schizophrenia and sex differences in heritability. *Mol. Psychiatry* **23**, 666–673 (2018). 28439101 10.1038/mp.2017.77 PMC5696105

[5] Gelernter, J. et al. Genome-wide association study of post-traumatic stress disorder reexperiencing symptoms in >165,000 US veterans. *Nat. Neurosci.* **22**, 1394–1401 (2019). 31358989 10.1038/s41593-019-0447-7 PMC6953633

[6] Yehuda, R., Golier, J. A., Yang, R. K. & Tischler, L. Enhanced sensitivity to glucocorticoids in peripheral mononuclear leukocytes in posttraumatic stress disorder. *Biol. Psychiatry* **55**, 1110–1116 (2004). 15158431 10.1016/j.biopsych.2004.02.010

[7] Yehuda, R. et al. The cortisol and glucocorticoid receptor response to low dose dexamethasone administration in aging combat veterans and holocaust survivors with and without posttraumatic stress disorder. *Biol. Psychiatry* **52**, 393–403 (2002). 12242055 10.1016/s0006-3223(02)01357-4

[8] Somvanshi, P. R. et al. Role of enhanced glucocorticoid receptor sensitivity in inflammation in PTSD: insights from computational model for circadian-neuroendocrine-immune interactions. *Am. J. Physiol. Endocrinol. Metab.* **319**, E48–E66 (2020). 32315214 10.1152/ajpendo.00398.2019

[9] Breen, M. S. et al. Gene networks specific for innate immunity define post-traumatic stress disorder. *Mol. Psychiatry* **20**, 1538–1545 (2015). 25754082 10.1038/mp.2015.9 PMC4565790

[10] Daskalakis, N. P., Cohen, H., Cai, G., Buxbaum, J. D. & Yehuda, R. Expression profiling associates blood and brain glucocorticoid receptor signaling with trauma-related individual differences in both sexes. *Proc. Natl Acad. Sci. USA* **111**, 13529–13534 (2014). 25114262 10.1073/pnas.1401660111 PMC4169965

[11] Yehuda, R. et al. Lower methylation of glucocorticoid receptor gene promoter 1F in peripheral blood of veterans with posttraumatic stress disorder. *Biol. Psychiatry* **77**, 356–364 (2015). 24661442 10.1016/j.biopsych.2014.02.006

[12] Cathomas, F., Murrough, J. W., Nestler, E. J., Han, M. H. & Russo, S. J. Neurobiology of resilience: interface between mind and body. *Biol. Psychiatry* **86**, 410–420 (2019). 31178098 10.1016/j.biopsych.2019.04.011 PMC6717018

[13] Lorsch, Z. S. et al. Stress resilience is promoted by a Zfp189-driven transcriptional network in prefrontal cortex. *Nat. Neurosci.* **22**, 1413–1423 (2019). 31427770 10.1038/s41593-019-0462-8 PMC6713580

[14] Popoli, M., Yan, Z., McEwen, B. S. & Sanacora, G. The stressed synapse: the impact of stress and glucocorticoids on glutamate transmission. *Nat. Rev. Neurosci.* **13**, 22–37 (2011). 22127301 10.1038/nrn3138 PMC3645314

[15] Lehrner, A. et al. A randomized, double-blind, placebo-controlled trial of hydrocortisone augmentation of prolonged exposure for PTSD in US combat veterans. *Behav. Res. Ther.* **144**, 103924 (2021). 34298438 10.1016/j.brat.2021.103924

[16] Golier, J. A. et al. A pilot study of mifepristone in combat-related PTSD. *Depress. Res. Treat.* **2012**, 393251 (2012). 22611490 10.1155/2012/393251 PMC3348629

[17] Averill, L. A. et al. Glutamate dysregulation and glutamatergic therapeutics for PTSD: evidence from human studies. *Neurosci. Lett.* **649**, 147–155 (2017). 27916636 10.1016/j.neulet.2016.11.064 PMC5482215

[18] Fernando, M. B., Ahfeldt, T. & Brennand, K. J. Modeling the complex genetic architectures of brain disease. *Nat. Genet.* **52**, 363–369 (2020). 32203467 10.1038/s41588-020-0596-3 PMC7909729

[19] Lieberman, R., Kranzler, H. R., Levine, E. S. & Covault, J. Examining FKBP5 mRNA expression in human iPSC-derived neural cells. *Psychiatry Res* **247**, 172–181 (2017). 27915167 10.1016/j.psychres.2016.11.027 PMC5191911

[20] Cruceanu, C. et al. Cell-type-specific impact of glucocorticoid receptor activation on the developing brain: a cerebral organoid study. *Am. J. Psychiatry* **179**, 375–387 (2022). 34698522 10.1176/appi.ajp.2021.21010095

[21] Aden, P. et al. Glucocorticoids dexamethasone and hydrocortisone inhibit proliferation and accelerate maturation of chicken cerebellar granule neurons. *Brain Res.* **1418**, 32–41 (2011). 21925649 10.1016/j.brainres.2011.08.053

[22] Levone, B, R. et al. Adult-born neurons from the dorsal, intermediate, and ventral regions of the longitudinal axis of the hippocampus exhibit differential sensitivity to glucocorticoids. *Mol. Psychiatry* **26**, 3240–3252 (2021). 32709996 10.1038/s41380-020-0848-8

[23] Breen, M. S. et al. Differential transcriptional response following glucocorticoid activation in cultured blood immune cells: a novel approach to PTSD biomarker development. *Transl. Psychiatry* **9**, 201 (2019). 31434874 10.1038/s41398-019-0539-x PMC6704073

[24] Crabtree, G. R., Munck, A. & Smith, K. A. Glucocorticoids inhibit expression of Fc receptors on the human granulocytic cell line HL-60. *Nature* **279**, 338–339 (1979). 450087 10.1038/279338a0

[25] Odaka, H., Adachi, N. & Numakawa, T. Impact of glucocorticoid on neurogenesis. *Neural Regen. Res.* **12**, 1028–1035 (2017). 28852377 10.4103/1673-5374.211174 PMC5558474

[26] Cain, D. W. & Cidlowski, J. A. Immune regulation by glucocorticoids. *Nat. Rev. Immunol.* **17**, 233–247 (2017). 28192415 10.1038/nri.2017.1 PMC9761406

[27] Girgenti, M. J. et al. Transcriptomic organization of the human brain in post-traumatic stress disorder. *Nat. Neurosci.* **24**, 24–33 (2021). 33349712 10.1038/s41593-020-00748-7

[28] Schrode, N. et al. Synergistic effects of common schizophrenia risk variants. *Nat. Genet.* **51**, 1475–1485 (2019). 31548722 10.1038/s41588-019-0497-5 PMC6778520

[29] Wang, M. et al. Transformative network modeling of multi-omics data reveals detailed circuits, key regulators, and potential therapeutics for Alzheimer’s disease. *Neuron* **109**, 257–272 e214 (2021). 33238137 10.1016/j.neuron.2020.11.002 PMC7855384

[30] Flaherty, E. et al. Neuronal impact of patient-specific aberrant NRXN1alpha splicing. *Nat. Genet.* **51**, 1679–1690 (2019). 31784728 10.1038/s41588-019-0539-z PMC7451045

[31] Ho, S. M. et al. Rapid Ngn2-induction of excitatory neurons from hiPSC-derived neural progenitor cells. *Methods* **101**, 113–124 (2016). 26626326 10.1016/j.ymeth.2015.11.019 PMC4860098

[32] Pak, C. et al. Cross-platform validation of neurotransmitter release impairments in schizophrenia patient-derived NRXN1-mutant neurons. *Proc. Natl Acad. Sci. USA* **118**, e2025598118 (2021). 34035170 10.1073/pnas.2025598118 PMC8179243

[33] Marro, S. G. et al. Neuroligin-4 regulates excitatory synaptic transmission in human neurons. *Neuron* **103**, 617–626 e616 (2019). 31257103 10.1016/j.neuron.2019.05.043 PMC6706319

[34] Zhang, Z. et al. The fragile X mutation impairs homeostatic plasticity in human neurons by blocking synaptic retinoic acid signaling. *Sci. Transl. Med.* **10**, eaar4338 (2018). 30068571 10.1126/scitranslmed.aar4338 PMC6317709

[35] Yi, F. et al. Autism-associated SHANK3 haploinsufficiency causes Ih channelopathy in human neurons. *Science* **352**, aaf2669 (2016). 26966193 10.1126/science.aaf2669 PMC4901875

[36] Zhang, Y. et al. Rapid single-step induction of functional neurons from human pluripotent stem cells. *Neuron* **78**, 785–798 (2013). 23764284 10.1016/j.neuron.2013.05.029 PMC3751803

[37] Meijer, M. et al. A single-cell model for synaptic transmission and plasticity in Human iPSC-derived neurons. *Cell Rep.* **27**, 2199–2211 e2196 (2019). 31091456 10.1016/j.celrep.2019.04.058

[38] Zhang, S. et al. Allele-specific open chromatin in human iPSC neurons elucidates functional disease variants. *Science* **369**, 561–565 (2020). 32732423 10.1126/science.aay3983 PMC7773145

[39] Sun, Y. et al. A deleterious Nav1.1 mutation selectively impairs telencephalic inhibitory neurons derived from Dravet syndrome patients. *eLife* **5**, e13073 (2016). 27458797 10.7554/eLife.13073 PMC4961470

[40] Hoffman, G. E. et al. Transcriptional signatures of schizophrenia in hiPSC-derived NPCs and neurons are concordant with post-mortem adult brains. *Nat. Commun.* **8**, 2225 (2017). 29263384 10.1038/s41467-017-02330-5 PMC5738408

[41] Inoue, M. & Kuriyama, H. Glucocorticoids inhibit acetylcholine-induced current in chromaffin cells. *Am. J. Physiol.* **257**, C906–C912 (1989). 2596584 10.1152/ajpcell.1989.257.5.C906

[42] Schoepe, S., Schacke, H., May, E. & Asadullah, K. Glucocorticoid therapy-induced skin atrophy. *Exp. Dermatol.* **15**, 406–420 (2006). 16689857 10.1111/j.0906-6705.2006.00435.x

[43] Sanchez-Resendis, O. et al. Glucocorticoid-cholinergic interactions in the dorsal striatum in memory consolidation of inhibitory avoidance training. *Front Behav. Neurosci.* **6**, 33 (2012). 22737110 10.3389/fnbeh.2012.00033 PMC3381328

[44] Xiang, Y. Y., Dong, H., Yang, B. B., Macdonald, J. F. & Lu, W. Y. Interaction of acetylcholinesterase with neurexin-1beta regulates glutamatergic synaptic stability in hippocampal neurons. *Mol. Brain* **7**, 15 (2014). 24594013 10.1186/1756-6606-7-15 PMC3973991

[45] Matthews, J. G., Ito, K., Barnes, P. J. & Adcock, I. M. Defective glucocorticoid receptor nuclear translocation and altered histone acetylation patterns in glucocorticoid-resistant patients. *J. Allergy Clin. Immunol.* **113**, 1100–1108 (2004). 15208591 10.1016/j.jaci.2004.03.018

[46] Liston, C. & Gan, W. B. Glucocorticoids are critical regulators of dendritic spine development and plasticity in vivo. *Proc. Natl Acad. Sci. USA* **108**, 16074–16079 (2011). 21911374 10.1073/pnas.1110444108 PMC3179117

[47] Breen, M. S. et al. PTSD blood transcriptome mega-analysis: Shared inflammatory pathways across biological sex and modes of trauma. *Neuropsychopharmacology* **43**, 469–481 (2018). 28925389 10.1038/npp.2017.220 PMC5770765

[48] Rosso, I. M. et al. Insula and anterior cingulate GABA levels in posttraumatic stress disorder: preliminary findings using magnetic resonance spectroscopy. *Depress. Anxiety* **31**, 115–123 (2014). 23861191 10.1002/da.22155 PMC3894264

[49] Moller, A. T., Backstrom, T., Nyberg, S., Sondergaard, H. P. & Helstrom, L. Women with PTSD have a changed sensitivity to GABA-A receptor active substances. *Psychopharmacology* **233**, 2025–2033 (2016). 25345735 10.1007/s00213-014-3776-y

[50] Geuze, E. et al. Reduced GABAA benzodiazepine receptor binding in veterans with post-traumatic stress disorder. *Mol. Psychiatry* **13**, 74–83, 73 (2008). 17667960 10.1038/sj.mp.4002054

[51] Jaffe, A. E. et al. Decoding shared versus divergent transcriptomic signatures across cortico-amygdala circuitry in PTSD and depressive disorders. *Am. J. Psychiatry* **179**, 673–686 (2022). 35791611 10.1176/appi.ajp.21020162 PMC10697016

[52] Huckins, L. M. et al. Polygenic regulation of PTSD severity and outcomes among World Trade Center responders. Preprint at *medRxiv*10.1101/2020.12.06.20244772 (2020).

[53] Brennand, K. et al. Phenotypic differences in hiPSC NPCs derived from patients with schizophrenia. *Mol. Psychiatry* **20**, 361–368 (2015). 24686136 10.1038/mp.2014.22 PMC4182344

[54] Mariani, J. et al. Modeling human cortical development in vitro using induced pluripotent stem cells. *Proc. Natl Acad. Sci. USA* **109**, 12770–12775 (2012). 22761314 10.1073/pnas.1202944109 PMC3411972

[55] Pasca, A. M. et al. Functional cortical neurons and astrocytes from human pluripotent stem cells in 3D culture. *Nat. Methods* **12**, 671–678 (2015). 26005811 10.1038/nmeth.3415 PMC4489980

[56] Qian, X. et al. Brain-region-specific organoids using mini-bioreactors for modeling ZIKV exposure. *Cell* **165**, 1238–1254 (2016). 27118425 10.1016/j.cell.2016.04.032 PMC4900885

[57] Nicholas, C. R. et al. Functional maturation of hPSC-derived forebrain interneurons requires an extended timeline and mimics human neural development. *Cell. Stem Cell.* **12**, 573–586 (2013). 23642366 10.1016/j.stem.2013.04.005 PMC3699205

[58] Powell, S. K. *et al*. Induction of dopaminergic neurons for neuronal subtype-specific modeling of psychiatric disease risk. *Mol. Psychiatry*, 10.1038/s41380-021-01273-0 (2021). 10.1038/s41380-021-01273-0 PMC8898985 34493831

[59] Ninomiya, E. et al. Glucocorticoids promote neural progenitor cell proliferation derived from human induced pluripotent stem cells. *Springerplus* **3**, 527 (2014). 25279318 10.1186/2193-1801-3-527 PMC4174547

[60] Raciti, M. et al. Glucocorticoids alter neuronal differentiation of human neuroepithelial-like cells by inducing long-lasting changes in the reactive oxygen species balance. *Neuropharmacology* **107**, 422–431 (2016). 26992751 10.1016/j.neuropharm.2016.03.022

[61] Provencal, N. et al. Glucocorticoid exposure during hippocampal neurogenesis primes future stress response by inducing changes in DNA methylation. *Proc. Natl Acad. Sci. USA* **117**, 23280–23285 (2020). 31399550 10.1073/pnas.1820842116 PMC7519233

[62] Buss, C. et al. Intergenerational transmission of maternal childhood maltreatment exposure: implications for fetal brain development. *J. Am. Acad. Child Adolesc. Psychiatry* **56**, 373–382 (2017). 28433086 10.1016/j.jaac.2017.03.001 PMC5402756

[63] Carson, R., Monaghan-Nichols, A. P., DeFranco, D. B. & Rudine, A. C. Effects of antenatal glucocorticoids on the developing brain. *Steroids* **114**, 25–32 (2016). 27343976 10.1016/j.steroids.2016.05.012 PMC5052110

[64] Daskalakis, N. P., Rijal, C. M., King, C., Huckins, L. M. & Ressler, K. J. Recent genetics and epigenetics approaches to PTSD. *Curr. Psychiatry Rep.* **20**, 30 (2018). 29623448 10.1007/s11920-018-0898-7 PMC6486832

[65] Yehuda, R. et al. Putative biological mechanisms for the association between early life adversity and the subsequent development of PTSD. *Psychopharmacology (Berl.)* **212**, 405–17 (2010). 20706708 10.1007/s00213-010-1969-6

[66] Stevens, J. S. & Jovanovic, T. Role of social cognition in post-traumatic stress disorder: a review and meta-analysis. *Genes Brain Behav.* **18**, e12518 (2019). 30221467 10.1111/gbb.12518 PMC6342618

[67] Velikonja, T., Fett, A. K. & Velthorst, E. Patterns of nonsocial and social cognitive functioning in adults with autism spectrum disorder: a systematic review and meta-analysis. *JAMA Psychiatry* **76**, 135–151 (2019). 30601878 10.1001/jamapsychiatry.2018.3645 PMC6439743

[68] de Kloet, C. S. et al. Enhanced cortisol suppression in response to dexamethasone administration in traumatized veterans with and without posttraumatic stress disorder. *Psychoneuroendocrinology* **32**, 215–226 (2007). 17296270 10.1016/j.psyneuen.2006.12.009

[69] OMIM. *Online Mendelian Inheritance in Man: An Online Catalog of Human Genes and Genetic Disorders*, https://omim.org/ (2021).

[70] Firth, H. V. et al. DECIPHER: Database of chromosomal imbalance and phenotype in Humans using ensembl resources. *Am. J. Hum. Genet* **84**, 524–533 (2009). 19344873 10.1016/j.ajhg.2009.03.010 PMC2667985

[71] Gilman, S. R. et al. Rare de novo variants associated with autism implicate a large functional network of genes involved in formation and function of synapses. *Neuron* **70**, 898–907 (2011). 21658583 10.1016/j.neuron.2011.05.021 PMC3607702

[72] Buxbaum, J. D. et al. The autism sequencing consortium: large-scale, high-throughput sequencing in autism spectrum disorders. *Neuron* **76**, 1052–1056 (2012). 23259942 10.1016/j.neuron.2012.12.008 PMC3863639

[73] Betancur, C. Etiological heterogeneity in autism spectrum disorders: more than 100 genetic and genomic disorders and still counting. *Brain Res.* **1380**, 42–77 (2011). 21129364 10.1016/j.brainres.2010.11.078

[74] Abrahams, B. S. et al. SFARI Gene 2.0: a community-driven knowledgebase for the autism spectrum disorders (ASDs). *Mol. Autism* **4**, 36 (2013). 24090431 10.1186/2040-2392-4-36 PMC3851189

[75] Tylee, D. S. et al. Blood transcriptomic comparison of individuals with and without autism spectrum disorder: a combined-samples mega-analysis. *Am. J. Med Genet B Neuropsychiatr. Genet* **174**, 181–201 (2017). 27862943 10.1002/ajmg.b.32511 PMC5499528

[76] Yang, C. et al. AutismKB 2.0: a knowledgebase for the genetic evidence of autism spectrum disorder. *Database (Oxford)* **2018**, 10.1093/database/bay106 (2018). 10.1093/database/bay106 PMC6193446 30339214

[77] Parikshak, N. N. et al. Integrative functional genomic analyses implicate specific molecular pathways and circuits in autism. *Cell* **155**, 1008–1021 (2013). 24267887 10.1016/j.cell.2013.10.031 PMC3934107

[78] Pinto, D. et al. Functional impact of global rare copy number variation in autism spectrum disorders. *Nature* **466**, 368–372 (2010). 20531469 10.1038/nature09146 PMC3021798

[79] Hess, J. L. et al. Transcriptome-wide mega-analyses reveal joint dysregulation of immunologic genes and transcription regulators in brain and blood in schizophrenia. *Schizophr. Res* **176**, 114–124 (2016). 27450777 10.1016/j.schres.2016.07.006 PMC5026943

[80] Cocchi, E., Drago, A. & Serretti, A. Hippocampal pruning as a new theory of schizophrenia etiopathogenesis. *Mol. Neurobiol.* **53**, 2065–2081 (2016). 25902861 10.1007/s12035-015-9174-6

[81] Clifton, N. E. et al. Genetic association of FMRP targets with psychiatric disorders. *Mol. Psychiatry* **26**, 2977–2990 (2021). 33077856 10.1038/s41380-020-00912-2 PMC8505260

[82] American Psychiatric Association. *Diagnostic and statistical manual of mental disorders. 4th ed (DSM-IV*) (American Psychiatric Press, 1994).

[83] First, M. B., Spitzer, R. L., Gibbon, M. & Williams, J. B. *Structured Clinical Interview for DSM-5—Research Version (SCID-5 for DSM-5, Research Version; SCID-5-RV, v.1.0.0)*. (American Psychiatric Publishing, 2015).

[84] Blake, D. D. et al. The development of a clinician-administered PTSD scale. *J. Trauma Stress* **8**, 75–90 (1995). 7712061 10.1007/BF02105408

[85] Kahler, D. J. et al. Improved methods for reprogramming human dermal fibroblasts using fluorescence activated cell sorting. *PLoS ONE* **8**, e59867 (2013). 23555815 10.1371/journal.pone.0059867 PMC3612089

[86] Paull, D. et al. Automated, high-throughput derivation, characterization and differentiation of induced pluripotent stem cells. *Nat. Methods* **12**, 885–892 (2015). 26237226 10.1038/nmeth.3507 PMC13012702

[87] Breen, M. S. et al. Modeling gene x environment interactions in PTSD using glucocorticoid-induced transcriptomics in human neurons. Preprint at *bioRxiv*10.1101/2021.03.01.433391 (2021).

[88] Piechota, M. et al. Transcriptional signatures of steroid hormones in the striatal neurons and astrocytes. *BMC Neurosci.* **18**, 37 (2017). 28381250 10.1186/s12868-017-0352-5 PMC5381047

[89] Salvador, E., Shityakov, S. & Forster, C. Glucocorticoids and endothelial cell barrier function. *Cell Tissue Res.* **355**, 597–605 (2014). 24352805 10.1007/s00441-013-1762-z PMC3972429

[90] Schaffter, N. et al. Serum cortisol as a predictor for posttraumatic stress disorder symptoms in post-myocardial infarction patients. *J. Affect. Disord.* **292**, 687–694 (2021). 34157664 10.1016/j.jad.2021.05.065

[91] Reddy, T. E. et al. Genomic determination of the glucocorticoid response reveals unexpected mechanisms of gene regulation. *Genome Res.* **19**, 2163–2171 (2009). 19801529 10.1101/gr.097022.109 PMC2792167

[92] Bolger, A. M., Lohse, M. & Usadel, B. Trimmomatic: a flexible trimmer for Illumina sequence data. *Bioinformatics* **30**, 2114–2120 (2014). 24695404 10.1093/bioinformatics/btu170 PMC4103590

[93] Dobin, A. et al. STAR: ultrafast universal RNA-seq aligner. *Bioinformatics* **29**, 15–21 (2013). 23104886 10.1093/bioinformatics/bts635 PMC3530905

[94] Liao, Y., Smyth, G. K. & Shi, W. featureCounts: an efficient general purpose program for assigning sequence reads to genomic features. *Bioinformatics* **30**, 923–930 (2014). 24227677 10.1093/bioinformatics/btt656

[95] Ritchie, M. E. et al. limma powers differential expression analyses for RNA-sequencing and microarray studies. *Nucleic Acids Res.* **43**, e47 (2015). 25605792 10.1093/nar/gkv007 PMC4402510

[96] Hoffman, G. E. & Schadt, E. E. variancePartition: interpreting drivers of variation in complex gene expression studies. *BMC Bioinf.* **17**, 483 (2016). 10.1186/s12859-016-1323-z PMC5123296 27884101

[97] Willer, C. J., Li, Y. & Abecasis, G. R. METAL: fast and efficient meta-analysis of genomewide association scans. *Bioinformatics* **26**, 2190–2191 (2010). 20616382 10.1093/bioinformatics/btq340 PMC2922887

[98] Zhang, B. & Horvath, S. A general framework for weighted gene co-expression network analysis. *Stat. Appl. Genet. Mol. Biol.* **4**, 17 (2005). 10.2202/1544-6115.1128 16646834

[99] Chen, J., Bardes, E. E., Aronow, B. J. & Jegga, A. G. ToppGene suite for gene list enrichment analysis and candidate gene prioritization. *Nucleic Acids Res.* **37**, W305–W311 (2009). 19465376 10.1093/nar/gkp427 PMC2703978

[100] Szklarczyk, D. et al. STRING v10: protein-protein interaction networks, integrated over the tree of life. *Nucleic Acids Res.* **43**, D447–D452 (2015). 25352553 10.1093/nar/gku1003 PMC4383874

[101] Shannon, P. et al. Cytoscape: a software environment for integrated models of biomolecular interaction networks. *Genome Res.* **13**, 2498–2504 (2003). 14597658 10.1101/gr.1239303 PMC403769

[102] Piñero, J. et al. The DisGeNET knowledge platform for disease genomics: 2019 update. *Nucleic Acids Res.* **48**, D845–D855 (2019). 10.1093/nar/gkz1021 PMC7145631 31680165

[103] Wittenberg, G. M. et al. Major depressive disorder is associated with differential expression of innate immune and neutrophil-related gene networks in peripheral blood: a quantitative review of whole-genome transcriptional data from case-control studies. *Biol. Psychiatry* **88**, 625–637 (2020). 32653108 10.1016/j.biopsych.2020.05.006

[104] Satterstrom, F. K. et al. Large-scale exome sequencing study implicates both developmental and functional changes in the neurobiology of autism. *Cell* **180**, 568–584.e23 (2020). 31981491 10.1016/j.cell.2019.12.036 PMC7250485

[105] De Rubeis, S. et al. Synaptic transcriptional and chromatin genes disrupted in autism. *Nature* **515**, 209–215 (2014). 25363760 10.1038/nature13772 PMC4402723

[106] Pantazatos, S. P. et al. Whole-transcriptome brain expression and exon-usage profiling in major depression and suicide: evidence for altered glial endothelial and ATPase activity. *Mol. Psychiatry* **22**, 760–773 (2017). 27528462 10.1038/mp.2016.130 PMC5313378

[107] Fromer, M. et al. Gene expression elucidates functional impact of polygenic risk for schizophrenia. *Nat. Neurosci.* **19**, 1442–1453 (2016). 27668389 10.1038/nn.4399 PMC5083142

[108] Darnell, J. C. et al. FMRP stalls ribosomal translocation on mRNAs linked to synaptic function and autism. *Cell* **146**, 247–261 (2011). 21784246 10.1016/j.cell.2011.06.013 PMC3232425

[109] Boyle, E. I. et al. GO::TermFinder–open source software for accessing gene ontology information and finding significantly enriched gene ontology terms associated with a list of genes. *Bioinformatics* **20**, 3710–3715 (2004). 15297299 10.1093/bioinformatics/bth456 PMC3037731

[110] Wang, J. & Liao, Y. *WebGestaltR: Gene Set Analysis Toolkit WebGestaltR. R package version 0.4.3*. https://CRAN.R-project.org/package=WebGestaltR (2020).

[111] Kolde, R. *pheatmap: Pretty Heatmaps. R package version 1.0.12*. https://CRAN.R-project.org/package=pheatmap (2019).

[112] Gamazon, E. R. et al. A gene-based association method for mapping traits using reference transcriptome data. *Nat. Genet.* **47**, 1091–1098 (2015). 26258848 10.1038/ng.3367 PMC4552594

[113] Xie, X. et al. Systematic discovery of regulatory motifs in human promoters and 3′ UTRs by comparison of several mammals. *Nature* **434**, 338–345 (2005). 15735639 10.1038/nature03441 PMC2923337

[114] Watanabe, K., Taskesen, E., van Bochoven, A. & Posthuma, D. Functional mapping and annotation of genetic associations with FUMA. *Nat. Commun.* **8**, 1826 (2017). 29184056 10.1038/s41467-017-01261-5 PMC5705698

[115] Marchese, S. et al. Altered gene expression and PTSD symptom dimensions in World Trade Center responders. Preprint at *medRxiv*10.1101/2021.03.05.21252989 (2021). 10.1038/s41380-022-01457-2 35177824

[116] Mertens, J. et al. Differential responses to lithium in hyperexcitable neurons from patients with bipolar disorder. *Nature* **527**, 95–99 (2015). 26524527 10.1038/nature15526 PMC4742055

[117] Mariani, J. et al. FOXG1-dependent dysregulation of GABA/glutamate neuron differentiation in autism spectrum disorders. *Cell* **162**, 375–390 (2015). 26186191 10.1016/j.cell.2015.06.034 PMC4519016

