# Author Correction: Modeling gene × environment interactions in PTSD using human neurons reveals diagnosis-specific glucocorticoid-induced gene expression

**DOI:** 10.1038/s41593-024-01854-6

**Published:** 2024-12-04

**Authors:** Carina Seah, Michael S. Breen, Tom Rusielewicz, Heather N. Bader, Changxin Xu, Christopher J. Hunter, Barry McCarthy, P. J. Michael Deans, Mitali Chattopadhyay, Jordan Goldberg, Saunil Dobariya, Frank Desarnaud, Iouri Makotkine, Janine D. Flory, Linda M. Bierer, Migle Staniskyte, NYSCF Global Stem Cell Array® Team, Lauren Bauer, Katie Brenner, Geoff Buckley-Herd, Sean DesMarteau, Patrick Fenton, Peter Ferrarotto, Jenna Hall, Selwyn Jacob, Travis Kroeker, Gregory Lallos, Hector Martinez, Paul McCoy, Frederick J. Monsma, Dorota Moroziewicz, Reid Otto, Kathryn Reggio, Bruce Sun, Rebecca Tibbets, Dong Woo Shin, Hongyan Zhou, Matthew Zimmer, Scott A. Noggle, Laura M. Huckins, Daniel Paull, Kristen J. Brennand, Rachel Yehuda

**Affiliations:** 1 Pamela Sklar Division of Psychiatric Genomics, Department of Psychiatry or Department of Genetics and Genomic Sciences, Icahn School of Medicine at Mount Sinai, New York, NY USA; 2 Nash Family Department of Neuroscience or Friedman Brain Institute, Icahn School of Medicine at Mount Sinai, New York, NY USA; 3 Departments of Psychiatry and Genetics, Division of Molecular Psychiatry, Yale University School of Medicine, New Haven, CT USA; 4 The New York Stem Cell Foundation Research Institute, New York, NY USA; 5 James J. Peters Veterans Affairs Medical Center, Bronx, NY USA; 6 Center for Psychedelic Psychotherapy and Trauma Research, Icahn School of Medicine at Mount Sinai, New York, NY USA

**Keywords:** Gene expression profiling, Post-traumatic stress disorder

Correction to: *Nature Neuroscience* 10.1038/s41593-022-01161-y, published online 20 October 2022.

In the version of the article initially published, Saunil Dobariya (The New York Stem Cell Foundation Research Institute, New York, NY, USA) was missing from the author list and is now included in the HTML and PDF versions of the article.

