# Supplementary material for: Modeling gene × environment interactions in PTSD using human neurons reveals diagnosis-specific glucocorticoid-induced gene expression
Source: Nat Neurosci. 2022 Oct 20;25(11):1434–45. doi: 10.1038/s41593-022-01161-y (PMC9630117; doi:10.1038/s41593-022-01161-y)
Supplement: Supplementary file 1 — Reporting Summary [file 41593_2022_1161_MOESM1_ESM.pdf]

nature portfolio

Corresponding author(s): Kristen Brennand

Last updated by author(s): 07/27/2022

Reporting Summary

Nature Portfolio wishes to improve the reproducibility of the work that we publish. This form provides structure for consistency and transparency in reporting. For further information on Nature Portfolio policies, see our Editorial Policies and the Editorial Policy Checklist.

Statistics

For all statistical analyses, confirm that the following items are present in the figure legend, table legend, main text, or Methods section.

n/a	Confirmed	
☐	☒	The exact sample size (*n*) for each experimental group/condition, given as a discrete number and unit of measurement
☐	☒	A statement on whether measurements were taken from distinct samples or whether the same sample was measured repeatedly
☐	☒	The statistical test(s) used AND whether they are one- or two-sided<br>*Only common tests should be described solely by name; describe more complex techniques in the Methods section.*
☐	☒	A description of all covariates tested
☐	☒	A description of any assumptions or corrections, such as tests of normality and adjustment for multiple comparisons
☐	☒	A full description of the statistical parameters including central tendency (e.g. means) or other basic estimates (e.g. regression coefficient) AND variation (e.g. standard deviation) or associated estimates of uncertainty (e.g. confidence intervals)
☐	☒	For null hypothesis testing, the test statistic (e.g. F, t, r) with confidence intervals, effect sizes, degrees of freedom and P value noted<br>*Give P values as exact values whenever suitable.*
☒	☐	For Bayesian analysis, information on the choice of priors and Markov chain Monte Carlo settings
☒	☐	For hierarchical and complex designs, identification of the appropriate level for tests and full reporting of outcomes
☐	☒	Estimates of effect sizes (e.g. Cohen's *d*, Pearson's *r*), indicating how they were calculated

Our web collection on statistics for biologists contains articles on many of the points above.

Software and code

Policy information about availability of computer code

Data collection	Glutamatergic NGN2-neurons were generated from hiPSCs in two batches (batch 1 n=15 vs 15; batch 2 n=4 vs 5). For NGN2-neurons, RNA was harvested with RNeasy plus micro kit (Qiagen). A low-input RNA-sequencing protocol was applied for the generation of RNA-sequencing data from NGN2-neurons. No software was used for data collection.
Data analysis	All RNA-sequencing FASTQ files underwent matching analytical procedures. In brief, adapters were trimmed using Trim Galore, mapped using STAR v2.7.2a, counted using featureCounts from RSubRead v2.0.3. Data was normalized using VOOM (Limma) v3.22.7, variance quantified using variancePartition v1.27.3, tested for differential expression using limma, and co-expression using WGCNA v1.71. Extensions of WCGNA were applied to implement preservation-based summary statistics. Meta-analysis was performed using METAL. Protein-protein interactions were mapped using STRING v11.5, and visualized using cytoscape v3.8.0. Gene set enrichment analysis was performed using WebGestaltR v0.4.4. Original code generated for analyses in this paper are published here: https://github.com/BreenMS

For manuscripts utilizing custom algorithms or software that are central to the research but not yet described in published literature, software must be made available to editors and reviewers. We strongly encourage code deposition in a community repository (e.g. GitHub). See the Nature Portfolio guidelines for submitting code & software for further information.

Data

Policy information about availability of data

All manuscripts must include a data availability statement. This statement should provide the following information, where applicable:

- Accession codes, unique identifiers, or web links for publicly available datasets
- A description of any restrictions on data availability
- For clinical datasets or third party data, please ensure that the statement adheres to our policy

All raw RNA-seq FASTQ files have been uploaded to the gene expression omnibus under accession number XXXXX (will release following manuscript publication).

Field-specific reporting

Please select the one below that is the best fit for your research. If you are not sure, read the appropriate sections before making your selection.

☒ Life sciences ☐ Behavioural & social sciences ☐ Ecological, evolutionary & environmental sciences

For a reference copy of the document with all sections, see nature.com/documents/nr-reporting-summary-flat.pdf

Life sciences study design

All studies must disclose on these points even when the disclosure is negative.

Sample size	A total of 49 individuals were recruited to yield well-matched and largely over-lapping (30 shared individuals) hiPSC and PBMC cohorts, comprised of combat veterans with (n=19 hiPSC donors and n=20 PBMC donors) and without (n=20 hiPSC donors and n=20 PBMC donors)) PTSD. Sample sizes for this study were chosen based on comparisons to other idiopathic designs. Using an isogenic within-donor design, we were adequately powered to resolve Hcort specific effects. No statistical method was used to predetermine sample size for case-control comparisons
Data exclusions	We only include transcriptomic profiles from NGN2-neurons where donor genotype and RNA-seq were confirmed to match. All RNA-seq expression values were converted to log2 RPKM and subjected to unsupervised principal component analysis (PCA) to identify and remove outlier samples that lay outside 95% confidence intervals from the grand averages. This identified two outlier samples in NGN2-neuron batch 1 and one outlier sample in batch 2 that were excluded from our analysis.
Replication	For technical reasons, glucocorticoid treatment of NGN2-neurons (batch 1 n=15vs15, batch 2 n=4vs5) and PBMCs (batch A n=10vs10, batch B n=10vs10 ref23) were completed independently and then meta-analyzed together to adjust for batch effects. All attempts at replication were successful.
Randomization	We randomized the RNA-sequencing of the current study to ensure data was not in batches. We randomized around three blocking factors - diagnosis, concentration, and cell type. We randomized diagnosis across plates, but overall plate effect was also adjusted through residualization and stratification and meta-analysis.
Blinding	Following stem cell reprogramming, the clinical origin of each sample was blinded to those performing neuronal differentiations, RNA purification, and RNA sequencing, and analysis. Diagnosis was blinded over the course of all experiments including neuronal differentiations, imaging, and HCort treatment.

Reporting for specific materials, systems and methods

We require information from authors about some types of materials, experimental systems and methods used in many studies. Here, indicate whether each material, system or method listed is relevant to your study. If you are not sure if a list item applies to your research, read the appropriate section before selecting a response.

Materials & experimental systems

n/a	Involved in the study	
☐	☒	Antibodies
☐	☒	Eukaryotic cell lines
☒	☐	Palaeontology and archaeology
☒	☐	Animals and other organisms
☐	☒	Human research participants
☒	☐	Clinical data
☒	☐	Dual use research of concern

Methods

n/a	Involved in the study	
☒	☐	ChIP-seq
☒	☐	Flow cytometry
☒	☐	MRI-based neuroimaging

Antibodies

Antibodies used	Anti-NESTIN clone 10C2 (Millipore, MAB5326, 1:3000), Anti-MAP2 (Abcam, Ab5392, 1:3000)
Validation	Anti-NESTIN Millipore is validated for immunocytochemistry in neural stem cells. Anti-MAP2 Abcam is validated for immunocytochemistry in human.

Eukaryotic cell lines

Policy information about cell lines

Cell line source(s)	Cultured PBMCs and fibroblast-reprogrammed hiPSCs
Authentication	Samples were confirmed to key karyotypically normal using the Illumina Core Exome Genotyping Chip (Illumina, 20030770) and cnvPartition 3.2.0 (Illumina, Genome Studio). No cell lines displayed karyotypic abnormalities (no reported CNVs ?2.5 MB in size). All reported CNVs are shown in each certificate of analysis. hiPSC lines were confirmed to be viable post-thaw, achieving a minimum of 50% confluency within 10 days). Sample identity testing was performed using the SNPTrace assay, confirming correct sample association between parental fibroblast and hiPSC line. Gene expression analysis was using a custom Nanostring panel27 to confirm expression of pluripotency markers such as POU5F1, NANOG, and SOX2, and lack of expression of early differentiation markers such as AFP (Mesoderm), SOX17 (Endoderm), and NR2F2 (Ectoderm). A scorecard panel was used to confirm propensity to differentiate27. All hiPSC lines used in this study passed the above QC and have a certificate of analysis.
Mycoplasma contamination	As part of the hiPSC validation process, all samples were tested for the absence of Mycoplasma (Lonza, LT07-710) and confirmed to be sterile (Hardy Diagnostics, K82).
Commonly misidentified lines<br>(See ICLAC register)	n/a

Human research participants

Policy information about studies involving human research participants

Population characteristics	All participants reported a DSM-IV criterion A combat trauma24; all experienced deployment to active military combat zones and experienced one or more significant combat-related traumas. Individuals with and without PTSD did not have significant differences in childhood or pre-deployment trauma, deployment number or cumulative duration. Participants underwent psychological evaluation using the Structured Clinical Interview for DSM-5 (SCID)25 and the Clinician Administered PTSD Scale (CAPS)26 for determination of PTSD diagnosis and severity. Eligibility criteria and thresholds were based on CAPS for DSM-IV; PTSD(+) had a current CAPS-IV total score above 40 (frequency + intensity), whereas PTSD(-) participants were combat-exposed veterans had a total score below 40. Although DSM-IV criteria for PTSD were used for inclusion, note that PTSD+ participants also met criteria for PTSD based on DSM-5. Diagnostic and clinical exclusions included: i) presence of moderate or severe substance use disorder within the past 6 months, ii) lifetime history of primary psychotic or Bipolar I disorders, iii) self-reported history of moderate or severe traumatic brain injury, iv) neurological disorder or major systemic illness, v) treatment with systemic steroids, and for PTSD(−) only, vi) current or recurrent major depressive disorder. Psychotropic medication was permitted, but dosage stabilization for at least two weeks was required. ~20% of individuals across both groups are currently treated with psychiatric medications. Current oral steroid treatment was an exclusion based on the impact of systemic steroids on the HPA axis. Given the small sample size, there was no additional matching performed for clinical characteristics such as index trauma types, duration of the disorder, comorbidities, and psychiatric medications at time of recruitment. Available clinical information is summarized in Table 1 and presented in detail in Table S1. Average age of participants with PTSD was 25.3 and controls was 32.5. 100% of the cohort was male.
Recruitment	Research participants were recruited through clinical care centers at the Bronx VAMC as well as from other research studies being conducted by Dr. Yehuda's research team. Participants were also recruited through advertisements in local newspapers and on social media as well as through flyers and outreach to other Veteran organizations. A total of 49 individuals were recruited to yield well-matched and largely over-lapping (30 shared individuals) hiPSC and PBMC cohorts, comprised of combat veterans with (n=19 hiPSC donors and n=20 PBMC donors) and without (n=20 hiPSC donors and n=20 PBMC donors)) PTSD. Detailed information about donor breakdown in the hiPSC and PBMC studies, by experimental batch, in Table S1. Eligibility for participation was determined as previously described23. Participants were included serially in the order in which they consented until the enrollment targets were attained. It is possible that the recruitment process may have created a self-selection bias in that participants are actively seeking health treatment at VA or those who are willing to volunteer to participate in research studies.
Ethics oversight	Participants in this cross-sectional study were combat-exposed Operation Enduring Freedom, Operation Iraqi Freedom, and Operation New Dawn (OEF/OIF/OND) veterans with and without PTSD who provided written, informed consent. Approval of the protocol was performed by the veteran affairs human subjects committee and the Icahn School of Medicine at Mount Sinai (VA HS# YEH-16-03 and ISMMS HS#15-00886) and from whom a viable blood and/or fibroblast sample was provided and sufficient RNA for genome-wide expression analyses was extracted.

Note that full information on the approval of the study protocol must also be provided in the manuscript.
